# Impact of Glycoclustering on Stiffening of MUC5AC Peptides Revealed by High‐Efficiency Synthesis

**DOI:** 10.1002/anie.202508278

**Published:** 2025-09-13

**Authors:** Arseniy Galashov, Elisabetta di Gregorio, Polina Ponomareva, Marc Safferthal, Ekaterina Kazakova, Leïla Bechtella, Kevin Pagel, Marina Pigaleva, Benesh Joseph, Oliver Seitz

**Affiliations:** ^1^ Institut für Chemie Humboldt‐Universität zu Berlin Brook‐Taylor‐Straße 2 12489 Berlin Germany; ^2^ Institut für Experimentalphysik Freie Universität Berlin Arnimallee 14 14195 Berlin Germany; ^3^ Institut für Chemie und Biochemie Freie Universität Berlin Arnimallee 22 14195 Berlin Germany; ^4^ Institut für Chemie und Biochemie Freie Universität Berlin Altensteinstraße 23A 14195 Berlin Germany

**Keywords:** CD, Glycopeptides, Ligation, PELDOR/DEER, Solid‐phase synthesis

## Abstract

Clustered O‐glycosylation of long tandem repeat regions is the hallmark of secreted mucins such as MUC5AC. Glycosylation is thought to play a key role in rigidifying the peptide backbone. The synthesis of peptides containing extended O‐glycosylation clusters has proven challenging, thus limiting studies on the influence of glycoclustering on peptide structure. Here, we report an efficient glyco‐economic synthesis of peptides featuring a previously unattained degree of glycoclustering. The method is based on a fully automated, DMF‐free solid‐phase synthesis employing the solvent 1,3‐dioxolane (DOL) in all steps. The addition of Tween‐20 enabled fast couplings of and to GalNAcylated amino acids by using only 0.5 excess equivalents at room temperature. Five tandem repeats long MUC5AC glycopeptides containing up to 30 GalNAc residues (100% occupancy of potential glycosylation sites) were accessed by Diselenide–Selenoester Ligation and selective deselenization in the presence of terminal cysteine residues. Circular Dichroism (CD) measurements showed that progressive GalNAcylation shifts the conformational equilibrium from the random coil to the extended polyproline type II helix conformation. Pulsed Electron–Electron Double Resonance (PELDOR) spectroscopy measurements revealed a significant stiffening of the MUC5AC peptide backbone upon GalNAcylation of four or six amino acids in each octad repeat.

## Graphical abstract

Peptides featuring a previously unattained degree of glycoclustering were obtained by fully automated solid‐phase synthesis in 1,3‐dioxolane/Tween‐20 and diselenide–selenoester ligation. Circular Dichroism (CD) and Pulsed Electron‐Electron Double Resonance (PELDOR) measurements of densely GalNAcylated MUC5AC multi‐tandem repeat peptides provided insights into the correlation between the occupancy of O‐glycosylation sites and stiffening of the peptide backbone.

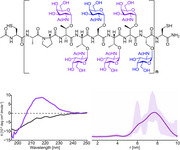

## Introduction

**Figure 1:**
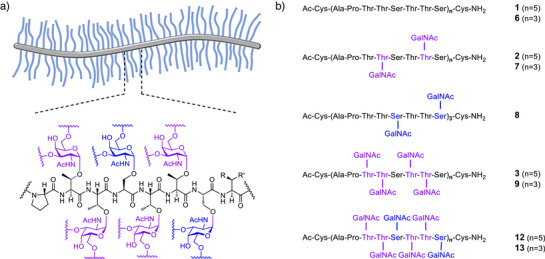
a) Schematic representation of the bottlebrush structure found in the PTS domains of mucins and the structure of a fully O‐glycosylated tandem repeat of MUC5AC. Most frequently R = R’ = H (alanine) or R = CH_3_, R’ = OH (threonine). b) α‐*O*‐GalNAcylated MUC5AC peptides targeted in this study.

Mucin glycoproteins are key components of the mucus layer that protects epithelial cells. Mucins differ from other glycoproteins by the presence of extended domains rich in tandem repeats of proline, threonine, and serine (PTS domains).^[^ ^1^, ^2^ ^]^ These regions serve as sites for O‐glycosylation and adopt an extended, rod‐like structure (Figure 1a). Glycosylation is believed to be crucial for hydrogelation.^[^ ^3^ ^]^ The O‐linked glycans enforce a “bottle‐brush” structure, protecting mucins against proteolytic cleavage and preventing them from collapsing into compact structures.^[^ ^4^ ^]^

The influence of O‐glycosylation on the 3D structure of mucins has been investigated, mainly by using nuclear magnetic resonance (NMR) studies on relatively short (6–10 AA) synthetic peptides.^[^ ^5^ ^]^ There is a consensus that α‐O‐GalNAcylation is required to stabilize the extended conformation of the peptide backbone.^[^ ^6^, ^7^, ^8^, ^9^ ^]^ However, conclusions about the structure of extended tandem repeat domains, which are based on an extrapolation, must be considered with caution because the conformational energy landscape of a peptide depends on its length.^[^ ^10^, ^11^ ^]^ Therefore, in the context of multi‐tandem repeats, it remains unclear how many of the numerous potential glycosylation sites must be occupied to confer a rod‐like structure on the glycopeptide. Although important for mucin function, the correlation between the occupancy of potential O‐glycosylation sites and structure has not been elucidated in the context of long multi‐tandem repeats, probably due to the difficulty synthesizing extended α‐O‐GalNAcylation clusters.

The previously available repertoire of synthesis methods was developed for the synthesis of mucin‐like peptides with up to approximately 40% O‐glycosylation degree.^[^ ^12^, ^13^ ^]^ However, many mucins have higher degrees of glycosylation. For example, MUC5AC can be glycosylated at each Ser/Thr residue of the multiply repeated PTTSTTSX consensus octads,^[^ ^14^ ^]^ where Ala and Thr are the most frequent amino acids at position X (Figure 1a, lower).^[^ ^15^ ^]^ This process results in tandem repeat regions, where 75%–87.5% of the amino acids carry α‐O‐linked GalNAc residues. Using the currently existing methods, the synthesis of fully α‐O‐GalNAcylated MUC5AC multi‐tandem repeats is extremely challenging.

Many labs have contributed to the advancement of the synthesis methodology, mainly to provide defined‐length mucin regions with specific glycan compositions for immunological studies.^[^ ^16^, ^17^, ^18^, ^19^, ^20^, ^21^, ^22^, ^23^, ^24^, ^25^, ^26^ ^]^ However, reports on the synthesis of peptides containing O‐glycosyl residues at several successive amino acids are scarce. It is partially due to the difficulty of coupling reactions with O‐linked glycoamino acids, which can undergo β‐elimination and are at risk of racemization.^[^ ^27^, ^28^ ^]^ Another contributing factor is the prohibitive cost of O‐GalNAcylated amino acids, which makes their application in high excess economically unfeasible during difficult couplings. Polymerization of Ser/Thr(αGalNAc)‐based *N*‐carboxyanhydrides allowed the preparation of densely GalNAcylated macromolecules.^[^ ^29^ ^]^ This method facilitates access to materials that can be used as mucin mimetics, which were characterized by atomic force microscopy (AFM). It was found that the persistence length is correlated with the degree of glycosylation. It is important to note that these glycopolymers do not contain the natural peptide sequence, and in particular, the absence of the structurally unique amino acid proline may have a significant impact.

The objective of the present study is to bridge the existing gap between NMR studies on short glycopeptides and AFM studies on long glycopolymers, to elucidate the impact of O‐GalNAcylation on the length and flexibility of peptides comprising multiple tandem repeats. Given its important role in defining the properties of mucus in both the gastrointestinal tract and the respiratory system,^[^ ^30^, ^31^ ^]^ we focused on the influence of multiple, site‐specific, clustered, and complete glycosylation of MUC5AC peptides. In order to achieve this objective, it was necessary to develop a flexible synthesis strategy that would allow for site‐specific labeling with reporter groups. Specifically, spin‐labeling would enable the determination of distances between the N‐ and C‐terminal ends in solution by pulsed electron‐electron double resonance (PELDOR, also known as DEER) spectroscopy.^[^ ^32^ ^]^

## Results and Discussion

### General Considerations

A set of nine MUC5AC glycopeptides was targeted with an aim to better understand how the number of tandem repeats, their degree of glycosylation, and glycosylation pattern affect stiffness/chain length (Figure 1b). Each peptide features specific glycosylation sites in varying arrangements and differing total quantity of glycans—0, 6, 10, 12, 18, 20, 30 GalNAcs. Peptides spanning three or five tandem repeats were targeted to evaluate the influence of peptide length on the structure. Using PELDOR, interspin distances can be determined in the 1.5–10 nm range under optimal conditions.^[^ ^33^, ^34^ ^]^ With the synthesis of glycopeptides **7** and **8**, we explored potential differences between glycosylation at threonine or serine without altering the total number of GalNAc moieties. Cysteine residues at the N‐ and the C‐terminal ends served as sites for the conjugation of spin labels.

### Highly Efficient and DMF‐Free Solid‐Phase Synthesis of MUC5AC Peptides

**Figure 2:**
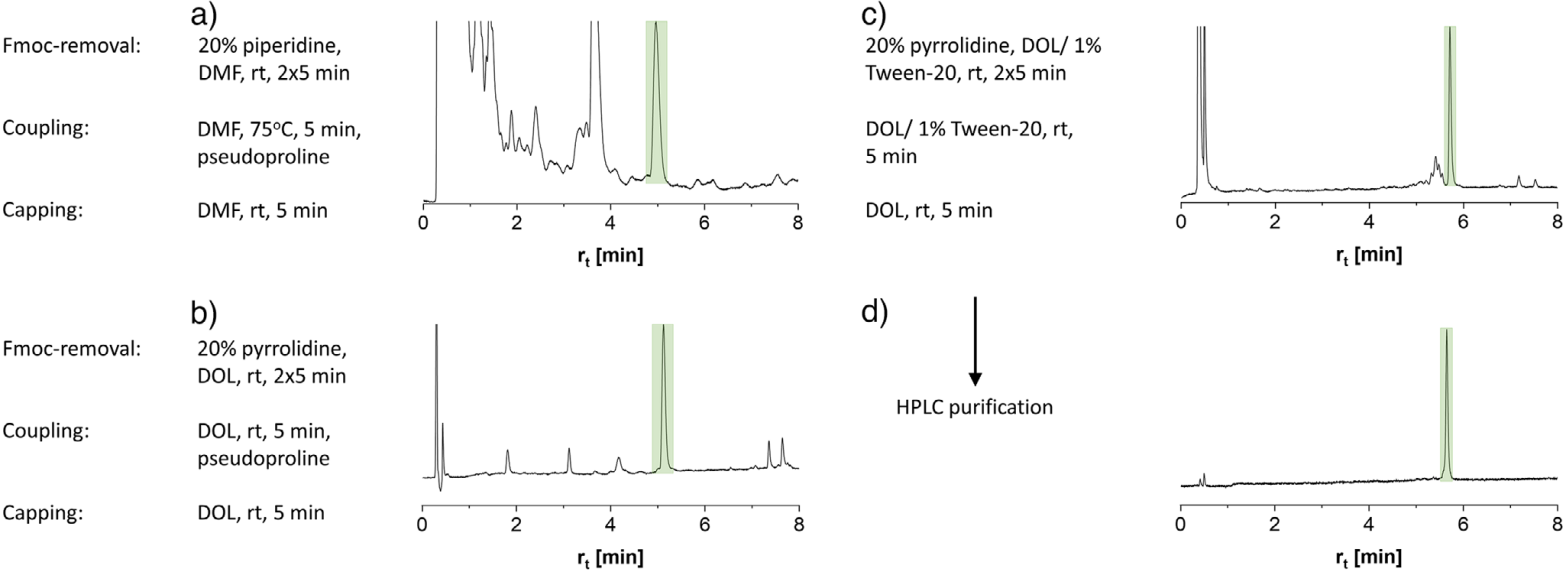
HPLC analysis of crude peptide **1** obtained after solid‐phase synthesis using a) DMF and pseudoproline in the first tandem repeat, b) DOL and pseudoproline, or c) DOL/1% Tween‐20 without pseudoproline instead of DMF. Green areas mark target compound. d) Peptide **1** after HPLC purification. Crude purity of A: 4%; B: 50%; C: 63%. The difference in retention times between A)/B) and C)/D) is due to a column exchange. Conditions: Coupling: 5 eqs. AA/DIC/Oxyma at 0.167 M concentration; Capping: Ac_2_O/diisopropylethylamine (DIPEA) (20%/10%) in solvent.

We commenced the work with the solid‐phase synthesis of a nonglycosylated peptide Ac‐C‐(APTTSTTS)_5_‐C (**1**). After coupling the C‐terminal cysteine residue to Tentagel R RAM resin, microwave irradiation was applied according to a recently reported method that demonstrated a highly efficient synthesis of β‐amyloid peptides.^[^ ^35^ ^]^ This method involved heating to 90 °C during Fmoc cleavage and coupling reactions involving *N*,*N’*‐diisopropylcarbodiimide (DIC) and ethyl cyano(hydroxylimino)acetate (Oxyma). To our surprise, we did not observe the target product during the HPLC‐MS analysis of crudes obtained after trifluoroacetic acid (TFA)‐induced cleavage (Figure S1A). According to a potentially milder MW‐SPPS method, temperatures were reduced to 75 °C, and Fmoc cleavage was performed at RT and prolonged. Again, we did not observe the target product (Figure S1B). It was described that sequences containing an abundance of threonines and/or serines could aggregate on resin, making the synthesis very challenging.^[^ ^36^ ^]^ While microwave irradiation is typically employed to overcome this problem, this was apparently insufficient for the 42 aa MUC5AC peptide **1**. Pseudoproline is known to prevent aggregation.^[^ ^37^ ^]^ Indeed, the introduction of pseudoproline in the C‐terminal tandem repeat – APTT**ST^psi^** TS provided the full‐length product, albeit at low purity (Figure 2a). While we expected improvements through the continued placement of pseudoproline residues, this approach is not feasible for the synthesis of fully glycosylated MUC5AC peptides. Instead, we considered a fundamental change in reaction conditions. A systematic investigation of the Schönleber and Pedersen groups had revealed an inverse correlation between solvent polarity and coupling efficiency.^[^ ^38^ ^]^ In previous work, we reported efficient solid‐phase syntheses of long mucin glycopeptides by using the rather nonpolar solvent 2‐MeTHF in rapid couplings of Fmoc‐Ser/Thr(αAc_3_GalNAc).^[^ ^39^ ^]^ However, the low viscosity of 2‐MeTHF (0.5 cP) prevents its use in commonly used synthesis robots. The necessity to perform manual couplings is a drawback, particularly when 75% of the amino acids are glycosylated. To enable a fully automated glycopeptide synthesis, we took inspiration from the Schönleber and Pedersen report, which described remarkably fast coupling reactions in 1,3‐dioxolane (DOL).^[^ ^38^ ^]^ Fortunately, the viscosity of DOL (0.6 cP) proved high enough to prevent solvent leakage from the reactor vial. Applied to the synthesis of the difficult MUC5AC tandem repeats, DOL was used in all steps, including Fmoc‐deprotection with pyrrolidine,^[^ ^40^ ^]^ coupling under activation of DIC/Oxyma, and capping. A direct comparison of the crude materials obtained after synthesis, including a single pseudoproline, demonstrates the significant enhancement achieved by substituting DMF with DOL (compare Figure 2b with 2a). Interestingly, even without recourse to pseudoproline, the purity of the crude full‐length peptide **1** was higher than for the synthesis in DMF with pseudoproline (39% vs. 4%, Figure S3). Seeking alternatives to the use of pseudoproline, we considered detergents as aggregation breakers. Non‐ionic detergents such as Triton X‐100 and Tween‐20 have been widely used to solubilize membrane proteins and as so‐called blocking agents in biochemical assays. In these applications, the agents function to prevent nonspecific interactions between proteins by adsorbing onto their surface. For Triton X‐100, it has been shown that this feature is helpful in preventing peptide aggregation during solid‐phase synthesis.^[^ ^41^ ^]^ However, Tween‐20 probably has lower toxicity considering its use as a food additive. Tween‐20 offers three potential hydrogen bond donor sites per molecule, in comparison to only one in Triton X‐100 (Figure S20). This could be advantageous in preventing peptide aggregation on a solid phase through interactions with the peptide amides. A literature survey suggested that Tween‐20 has not been used in solid‐phase peptide synthesis, perhaps surprisingly. Most remarkably, including 1% Tween‐20 during Fmoc deprotection and coupling resulted in a high purity (63%) of crude peptide **1** (Figure 2c). It is interesting to note that when Tween‐20 was used in conjunction with the DMF‐based MW‐SPPS method, the crude purity of peptide **1** remained low (3%, Figure S2). This can be attributed to the lower coupling rates in DMF compared to DOL.^[^ ^38^ ^]^ Regardless of the mechanism by which DOL/1% Tween‐20 enhances solid‐phase peptide synthesis, the results convinced us to explore the method in the synthesis of O‐glycopeptides.

### High Efficiency Fully Automated Solid‐Phase Synthesis of Densely GalNAcylated MUC5AC Peptides

**Scheme 1:**
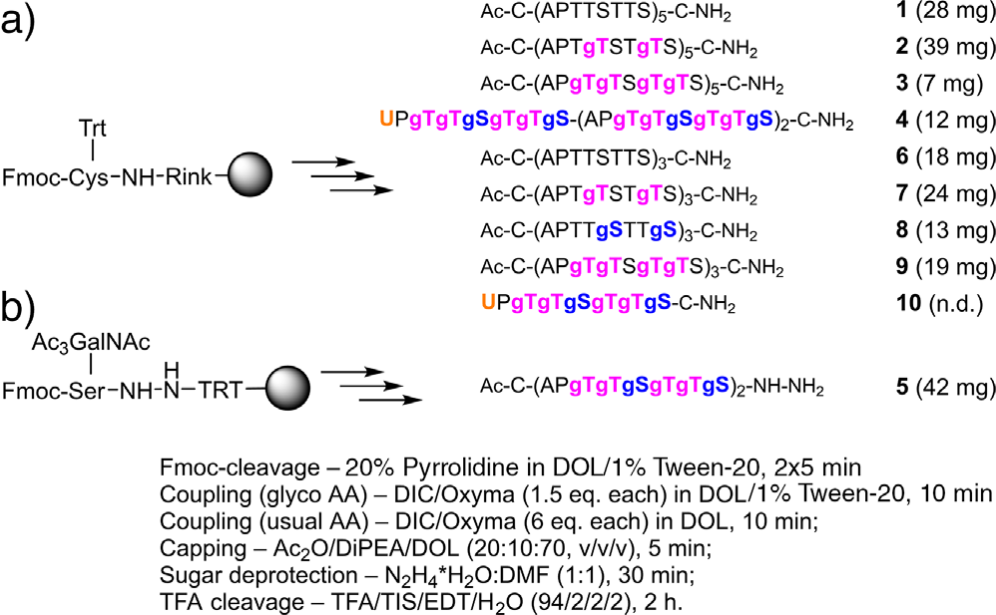
Solid‐phase synthesis in 40 µmol scale of MUC5AC peptides as C‐terminal a) amides and b) hydrazide. Amounts of material isolated after HPLC purification are given in parentheses. The position of Thr(αGalNAc)/Ser(αGalNAc) is indicated as gT/gS. Fmoc‐Ser(Trt)‐OH was used in the synthesis of **3** and **9**. Double coupling was performed for the last nine amino acids in **3**. Peptide **10** was not purified. (EDT, 1,2‐ethanedithiol; TIS, triisopropylsilane).

**Figure 3:**
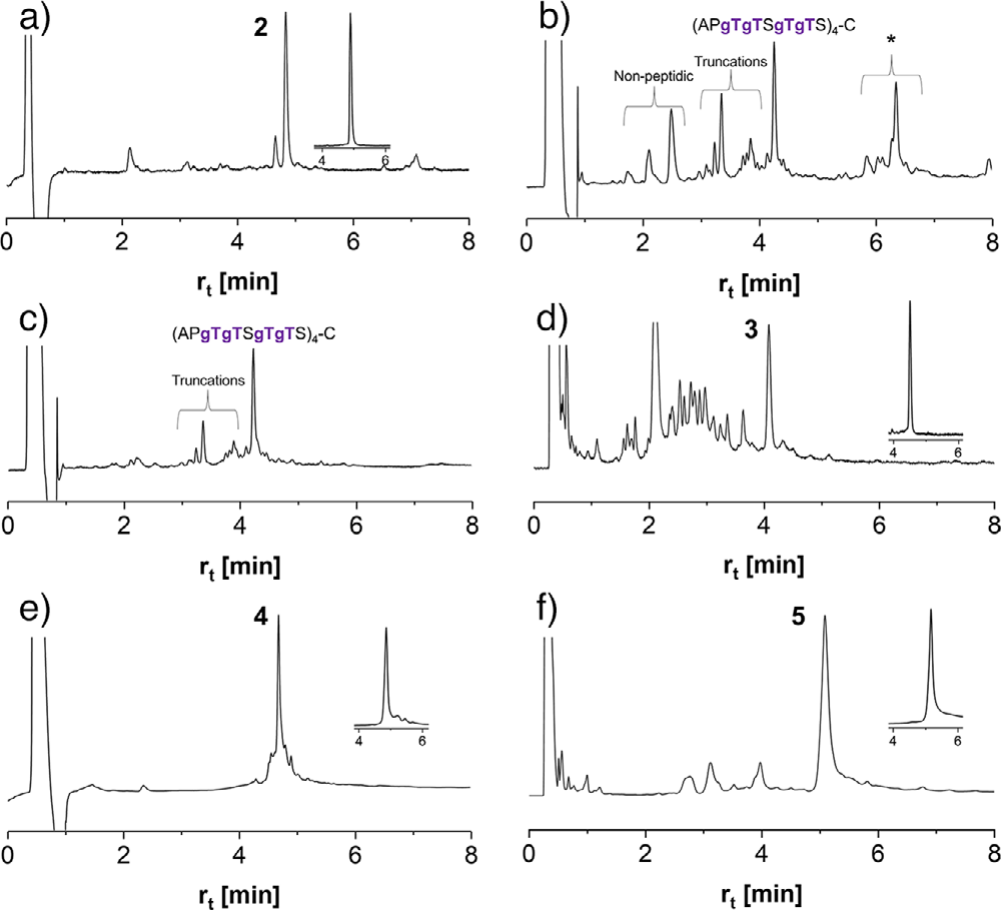
a)–f) HPLC analysis of crude glycopeptides. Inserts show HPLC analysis of purified glycopeptides. A) **2**, B) H‐(APgTgTSgTgTS)_4_‐NH_2_ obtained by using Fmoc‐Ser(*t*Bu) and TFA cleavage at room temp. or c) 40 °C. D) **3** obtained by using Fmoc‐Ser(Trt); D) **4** and F) **5**. Conditions: Solvent A (98.9% H_2_O + 0.1% MeCN + 0.1% TFA) and solvent B (98.9% MeCN + 0.1% H_2_O + 0.1% TFA) in gradients: 05–15% B in A for peptide **2**; 0%–10% B in A for peptides **3** and **4**; 2%–12% B in A for peptide **5,** and for crudes in B and C. *λ* = 210 nm.

Next, the DOL/Tween‐20‐based SPPS method was applied in the fully automated synthesis of α‐O‐GalNAcylated MUC5AC peptides (Scheme 1). As previously described,^[^ ^39^ ^]^ removal of O‐ acetyl protecting groups was performed on the solid phase by treatment with hydrazine hydrate before TFA cleavage. Surprisingly, despite using only 1.5 equivalents of Fmoc‐Thr(αAc_3_GalNAc) in the coupling reactions, the 5TR‐long glycopeptide **2** was obtained in higher purity than the non‐glycosylated peptide **1** (compare Figures 2c and 3a). This outcome may be related to the suppressed aggregation resulting from peptide glycosylation. However, difficulties became apparent when the number of O‐glycosylated amino acids was doubled from a total of 10 to 20 in glycopeptide **3**. After assembly of the fourth tandem repeat, HPLC–MS analysis of crude materials revealed truncations and nonpolar compounds with m/z values indicative of incomplete cleavage of the serine *tert*‐butyl (*t*Bu) ethers (Figure 3b), probably due to hindrance by the flanking glycosyl moieties. Reluctant *t*Bu removal reactions can be forced by performing TFA cleavage at 40 °C (Figure 3c); however, we chose to avoid harsh conditions and used *O*‐trityl protection (Fmoc‐Ser(Trt)‐OH instead of Fmoc‐Ser(*t*Bu)‐OH) in the subsequent syntheses. Yet, extension of the glycopeptide chain beyond three tandem repeats remained challenging, and amino acids were double‐coupled after the introduction of the fourth tandem repeat. The purity of the crude material was rather low, but due to the hydrophobicity introduced by the N‐terminal N‐acetyl‐cysteine, the 20‐fold GalNAcylated peptide **3** was easily separable by HPLC and obtained in high purity despite its > 50% glycosylation degree (Scheme 1, Figure 3d).

### Ligation of Densely O‐GalNAcylated Peptides

In light of the challenges encountered during the synthesis of glycopeptide **3**, it was anticipated that the synthesis of a fully GalNAcylated MUC5AC peptide (75% degree of glycosylation) spanning five tandem repeats would pose an even greater challenge. We, therefore, took recourse to a convergent approach based on native chemical ligation (NCL).^[^ ^42^ ^]^ The Ser(GalNAc)‐Ala bond is the only ligation junction that can be accessed directly by an NCL on cysteine and subsequent desulfurization.^[^ ^43^ ^]^ However, the terminal cysteine residues would have to be protected from desulfurization, as these are reserved for the introduction of spin labels. Diselenide‐selenoester ligation (DSL)^[^ ^44^ ^]^ at the seleno–cysteine glycopeptides **4** and **10** appeared to be a more promising approach since selenocysteine can be converted to alanine without affecting the unprotected cysteine side chains. First, the glycopeptide hydrazide **5** was targeted for later conversion to a glycopeptide selenoester. Fmoc‐Ser(αAc_3_GalNAc) was coupled to a hydrazide resin, and the following two tandem repeats were assembled in a fully automated solid‐phase synthesis. This process utilized only 1.5 equiv. of Fmoc‐Ser/Thr(αAc_3_GalNAc) in each coupling, and DOL/Tween‐20 as a replacement for DMF. The synthesis was remarkably efficient and provided crude material in high purity despite the need to create two clusters of six successive O‐ GalNAc residues (Figure 3f). For the synthesis of glycopeptides **4** and **10**, *N*‐Fmoc‐*Se*‐xanthyl‐protected selenocysteine was introduced after the assembly of one or three fully GalNAcylated MUC5AC tandem repeats. Purification required the presence of a reducing agent to prevent the formation of Se─Se and Se─S bonds. Despite the complications inherent to selenocysteine and three hexa‐GalNAc clusters, glycopeptide **4** was obtained in high purity (Figure 3e).

**Figure 4:**
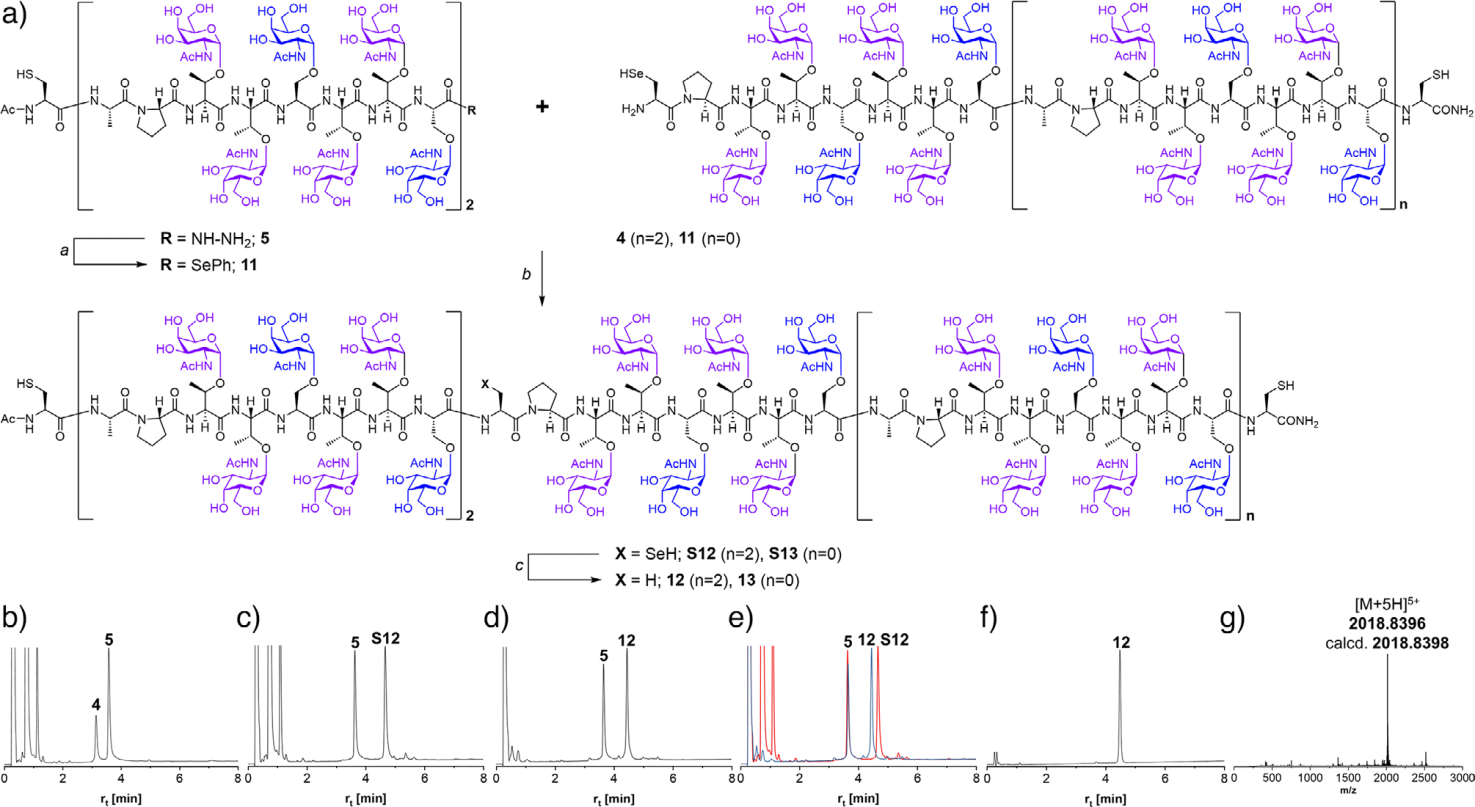
a) Convergent synthesis of glycopeptides **12** and **13**. Conditions: *a)* 1.5 equiv. acetylacetone, 10 mM Ph_2_Se_2_, 10 mM TCEP, 6 M Gd‐HCl, 0.2 M Na_2_HPO_4_, 37 °C, pH of 3.0, 56%; *b)* 10 mM **6**, 5 mM **4** or **11**, 6 M Gd‐HCl, 0.2 M Na_2_HPO_4_, pH 6; *c)* Hexane extraction, solution degassing, then: 2.5 mM **S12** or **S13**, 125 mM TCEP, 25 mM DTT, 22% (**12**) or 18% (**13**) yield after HPLC purification. Glycopeptide **11** was used without purification. HPLC analysis of B) the reaction between **4** and **11** at *t* = 0 min and C) *t* = 20 min, D) deselenization of **S12**, E) co‐injection of crude ligation product (see C)) and crude deselenization product (see D)), F) **12** after HPLC purification. G) ESI‐HRMS analysis of **12**. For analytical purposes, DTT and N_2_H_4_ were added to probes before HPLC measurements, resulting in conversion of unreacted **11** to **5**. (Gd‐HCl, guanidinium hydrochloride; DTT, dithiothreitol; TCEP, tris(2‐carboxyethyl)phosphine).

In preparation for DSL, glycopeptide hydrazide **5** was converted to the selenoester **11** (Figure 4a), as recently reported.^[^ ^45^ ^]^ HPLC analysis indicated that activation of the hydrazide with acetylacetone and treatment of the N‐acyl pyrazole intermediate formed with diphenyl diselenide/TCEP was unhindered by the presence of the O‐GalNAc residues (Figure S9). Subsequently, the 12‐fold GalNAcylated peptide selenoester **11** and the 18‐fold GalNAcylated selenocysteine glycopeptide **4** were allowed to react in the DSL at pH of 6. The reaction proceeded quickly and cleanly (Figure 4c). To drive the reaction to completion, two equiv. of **11** are needed (due to the formation of N‐ and double, N, Se‐acylated products). Without further purification, Ph_2_Se_2_ was extracted with hexane. TCEP and DTT were added to induce mild deselenization (Figure 4d). It was evidenced by the shift to a shorter retention time, which was expected due to the loss of a hydrophobic selenol (Figure 4e). The reaction cascade involving the formation of the glycopeptide selenoester, ligation, and deselenization proceeded remarkably smoothly, providing access to the MUC5AC peptide containing five fully O‐GalNAcylated tandem repeats without significant formation of by‐products. Due to this efficiency, the glycopeptide selenoester **11** and the selenocysteinyl glycopeptide **10** (Figure S8) were used in the DSL in non‐purified form, and the ligation product was submitted, again without purification, to deselenization. After a single HPLC purification, the densely O‐GalNAcylated peptide **13** was obtained in high purity (Figure S10).

### Circular Dichroism Studies

**Figure 5:**
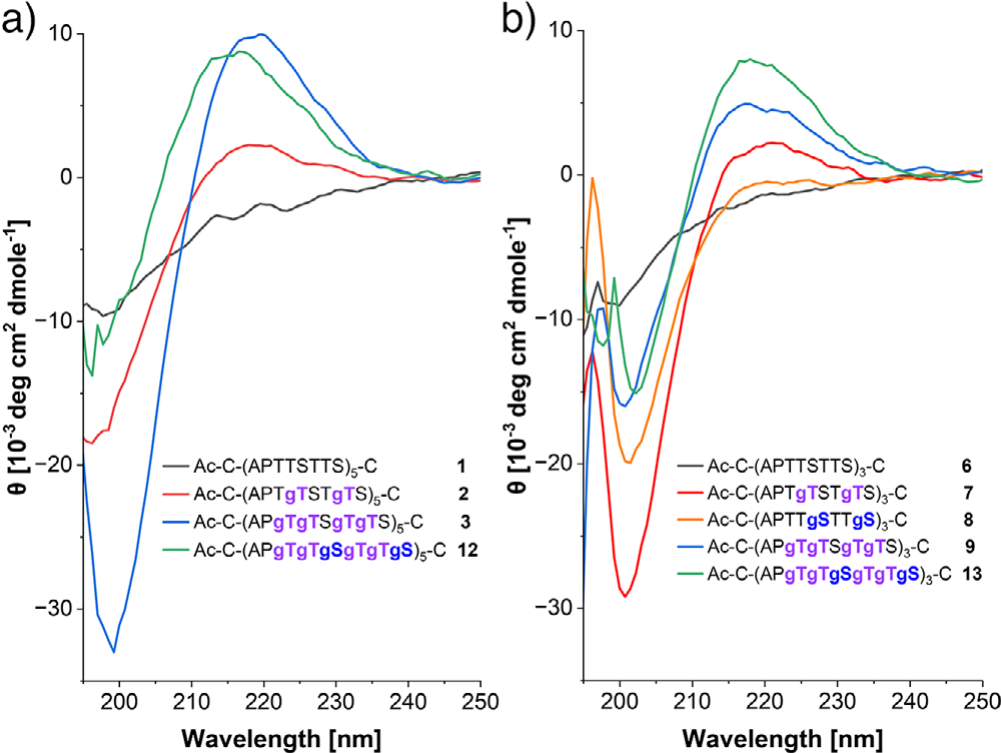
CD spectra of peptides containing a) five tandem repeats (**1–3**,**12**) and b) three tandem repeats (**6–9**, **13**). Conditions: 50 µM peptide, 150 mM NaCl, 25 mM Tris‐HCl, pH of 6.9. Spectra are corrected with calibration to pure GalNAc.

The CD signatures revealed fundamental differences in the secondary structures of glycosylated and non‐glycosylated MUC5AC multi‐tandem repeats (Figure 5). Non‐glycosylated peptides **1** and **6** exhibit characteristics of a random coil structure with a minimum at 198 nm.^[^ ^46^ ^]^ A maximum at 217 nm is observed when α‐O‐linked GalNAc moieties are appended to two threonine residues per tandem repeat in glycopeptide **2**. In addition, the minimum at 198 nm is more pronounced. The positive band at 217 nm is indicative of a polyproline type II (PPII) helix conformation, an extended backbone structure, which has previously been observed in polyprolines and collagen,^[^ ^47^ ^]^ antifreeze glycoproteins,^[^ ^48^, ^49^ ^]^ mucins^[^ ^8^, ^50^ ^]^ and mucin‐mimetic compounds.^[^ ^6^, ^29^, ^51^, ^52^ ^]^ As the GalNAc monosaccharide shows a weak negative band in this spectral region (Figure S11), the CD spectra of the glycopeptides (Figure S12) were corrected for its weak but noticeable contributions. In glycopeptide **3**, each of the four threonine residues per octad repeat is α‐O‐GalNAcylated, resulting in a profound increase of the maximum at 218 nm. This indicates a stabilization of the PPII conformation, which should result in a length extension. The PPII signature remained with the fully GalNAcylated peptide **12**, although a blue shift of the positive band and the increased molar ellipticity below 200 nm suggest that the two additional GalNAc residues slightly alter the conformational landscape. The analysis of glycopeptides comprised of three tandem repeats (**6**–**9**, **13**) revealed similar trends. Again, the PPII helix was stabilized as the number of α‐*O*‐GalNAc residues increased. Interestingly, a maximum at 220 nm was not observed when the serine residues were α‐O‐GalNAcylated in glycopeptide **8**. The two glycopeptides **7** and **8** have the same molecular weight and number of α‐O‐linked GalNAc residues per tandem repeat. However, the CD signatures indicate that, within the context of the MUC5AC tandem repeat, Ser(αGalNAc) is not as efficient in stabilizing the extended PPII helix conformation as Thr(αGalNAc). Considering the molar ellipticity at the 220 nm maximum, the fully GalNAcylated peptide **13** has the highest propensity to adopt the extended PPII helix. Two of the six GalNAc residues per octad repeat are linked to serine. We infer—the inability of Ser(GalNAc) to stabilize the PPII conformation of the MUC5AC tandem repeat cannot override the PPII stabilization induced by Thr(GalNAc).

### PELDOR Spectroscopy Measurements

**Figure 6:**
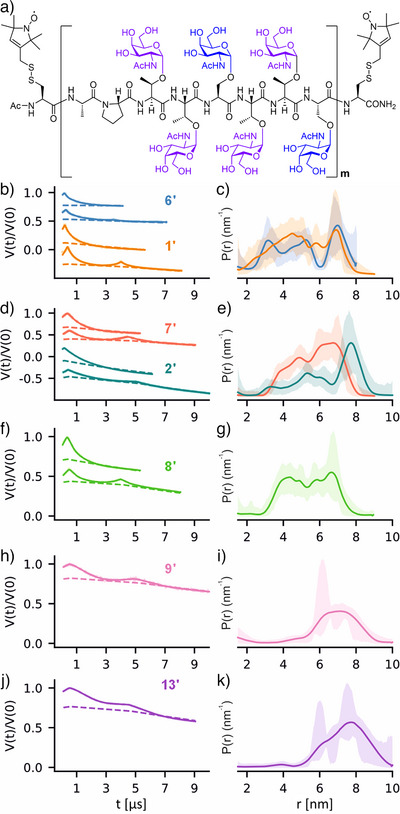
a) Representative example of MTSL‐labelled, multi‐GalNAcylated MUC5AC tandem repeats. b)–k) Primary 4‐pulse and/or 5‐pulse PELDOR data (left) and the corresponding distance distributions (right) determined using the DeerLab program.^[^ ^55^ ^]^ Data were globally analyzed for samples that included both 4‐pulse and 5‐puse PELDOR experiments. The error bounds indicate a 95% confidence interval. Conditions: 15–25 µM peptide, 150 mM NaCl, 25 mM Tris‐HCl + 15% deuterated glycerol (v/v).

To investigate their length and stiffness, the multi‐tandem repeat MUC5AC peptides were analyzed by PELDOR spectroscopy, which provides interspin distances by measuring dipolar couplings between unpaired electrons. MTSL (*S*‐(1‐oxyl‐2,2,5,5‐tetramethyl‐2,5‐dihydro‐1H‐pyrrol‐3‐yl)methyl methanesulfonothioate) was chosen to introduce spin labels to the N‐ and C‐terminal cysteines via disulfide bond formation (Figure 6a). After the one‐hour reaction and removal of residual MTSL by ultrafiltration or extraction with EtOAc, spin‐labeled peptides were obtained in high purity (Figure S13). As the ends of the peptides span a broad range of distances, we employed both 4‐pulse‐ and/or 5‐pulse PELDOR experiments to achieve more accurate distance measurements.^[^ ^53^, ^54^ ^]^

Distance distributions calculated from PELDOR spectra exhibited marked variation amongst the peptides under investigation. Both non‐glycosylated peptides (**1′** and **6′**) were found to sample a large conformational space, revealing a propensity for the ends of the peptides to arrange at a broad range of distances (Figure 6c). These non‐glycosylated peptides were measured at lower concentrations (15 µM) to exclude aggregation. It also limited the observable dipolar evolution time and, consequently, the upper limit of observable distances. The introduction of GalNAc sugars at two threonine residues per TR in glycopeptides **2′** and **7′** resulted in an overall shift of distributions towards longer distances, in particular for the longer glycopeptide **2′** (Figure 6e, the difference in the *r_max_* between the distributions is attributed to the difference in the observation window for the time domain data). At a total of four Thr‐linked GalNAc residues per TR in glycopeptides **9′**, the distance distribution (Figure 6i) indicated a markedly reduced propensity for the peptide ends to adopt distances smaller than 5 nm. The major peak centered around 7 nm dominating the distribution indicates an increased end‐to‐end distance, consistent with the stabilization of the PPII helix conformation observed by CD spectroscopy. The longer glycopeptide **3′** has the same number of Thr(GalNAc) residues per TR as **9′**, but the acquired time window of the PELDOR data (Figure S14) was insufficient to accurately determine the distances. We hypothesize that the clustered GalNAcylation of a 5‐TR MUC5AC peptide positions the two terminal spin labels at greater distances compared to the 3‐TR peptide. Subsequent measurements were, therefore, continued with the shorter peptides.

Given the different CD signatures, it was worthwhile to compare the distance distributions of glycopeptides containing α‐O‐linked GalNAc, either at two serine (**8′**) or two threonine (**7′**) residues per tandem repeat. However, the distributions appear similar (compare the trace for **7′** in Figure 6e with Figure 6g). Continuous wave EPR spectra, by contrast, revealed that the rotational correlation time determined for the paramagnetic reporter for the Ser(GalNAc) peptide **8′** is higher than for the Thr(GalNAc) peptide **7′** (Figure S16). This may be attributed to an increased propensity of **8′** to adopt bent conformations, which would localize the spin label close to the backbone where motions are hindered and subsequently affect the peptide conformation.

It is interesting to compare the fully GalNAcylated peptide **13′** with glycopeptide **9′** (Figure 6k vs. Figure 6i). For both glycopeptides, there is little propensity to arrange the terminal spin labels in distances shorter than 5 nm. The similarity of distance distributions suggests that GalNAcylation of the four threonine residues per tandem repeat is sufficient to stiffen the peptide backbone. Due to technical limitations for accurately measuring such long distances, it cannot be conclusively determined whether the end‐to‐end distance for **13** is longer than **9**.

## Discussion

A comparison of the PELDOR data shown in Figure 6 reveals an α*‐O*‐GalNAcylation‐induced stiffening of the MUC5AC peptide backbone. While nonglycosylated peptides were found to sample a large conformational space, the introduction of four or more GalNAc residues per TR induced substantial changes in the distribution of the end‐to‐end distance, as evidenced by a marked decrease in the propensity to adopt short end‐to‐end distances. This is consistent with a stiffening of the peptide backbone. In natural mucins, additional saccharides are attached to the 3‐*O* and 6‐*O* positions of the α‐*O*‐linked GalNAc core. The CD data suggests that the introduction of two or more α‐*O*‐linked GalNAc monosaccharide units to the MUC5AC tandem repeat is already sufficient to exhibit characteristics of a polyproline type II helix conformation provided that GalNAcylation occurs on threonine. In this respect, it is worth considering NMR studies that have highlighted the important role of threonine's β‐methyl group in limiting the conformational space available to the peptide backbone.^[^ ^56^, ^57^ ^]^ The methyl's occupancy of space has been reported to lock the GalNAc residue in a specific arrangement, while GalNAc on Ser has more degrees of freedom. While CD spectroscopy indicated that two Thr(GalNAc) but not the two Ser(GalNAc) residues per TR were capable of inducing features of a PPII helix conformation, the PELDOR data revealed that this was not sufficient to induce significant stiffening of the MUC5AC peptide backbone. Marked stiffening required GalNAcylation of all four Thr residues, i.e., 67% occupancy of all potential glycosylation sites. Considering the comparably small size of the GalNAc monosaccharide, the extent of stiffening is remarkable. With the distance distribution peaking at 7.7 nm and taking into account a 10–15 Å span of the MTSL labels, full GalNAcylation (glycopeptide **13′**) is estimated to provide the PPII conformation with a rise of approximately 2.5 Å per amino acid. For comparison, PELDOR measurements of oligoprolines suggest that each proline residue contributes a 2.7–3 Å to the length of the peptide.^[^ ^58^ ^]^ A 3.1 Å helical rise has been determined by NMR and crystal structure analyses for 3–13 residues long PPII segments of folded proteins.^[^ ^51^ ^]^

The PPII helix conformation is distinguished from other canonical secondary structures, such as the α‐helix or the β‐strand, by its openness and lack of intramolecular hydrogen bonds. In collagen single strands, *n*–π* interactions between the lone pairs of the proline carboxyl oxygen and amide bonds contribute to stabilization.^[^ ^59^ ^]^ Glycan structures have an organized hydration shell, and it is tempting to speculate that glycan‐bound water molecules interact with backbone amides. Therefore, it will be interesting to explore whether further stiffening/extension can be achieved through the introduction of more elaborated, branched glycans such as mucin core 2 and core 4 structures. Perhaps glycosylation with sterically demanding glycans will also enable serine to stabilize PPII conformations.

A key challenge associated with the synthesis of O‐glycopeptides is the high cost of glycoamino acid building blocks, which are typically introduced such that minimum amounts are required. It is standard practice to perform these couplings manually. We demonstrated that a highly efficient automated coupling is possible when the typically used DMF is replaced by 1% Tween‐20 in 1,3‐dioxolane (DOL). In fact, the solvent DOL can replace DMF, which is considered harmful to health and the environment, in every step of solid‐phase assembly. However, it should be noted that activators such as HATU/PyOxim/etc. are insoluble in DOL, which could also be the case for some amino acids. As Schönleber and Pedersen demonstrated, binary solvent mixtures can also be used.^[^ ^38^ ^]^ It is noteworthy that utilizing the DOL/Tween‐20 protocol with an excess of just 0.5 equiv. allows for the efficient coupling of two clusters of six consecutive GalNAc amino acids. However, we observed that further extensions are difficult. The incorporation of the “SynTag”, a strategy employed by the Hartrampf group to enhance the synthesis of challenging peptides, may offer benefits.^[^ ^60^ ^]^ However, at a certain length, by‐products of a linear solid‐phase synthesis cannot be easily separated from the target product. In such cases, a convergent synthesis strategy becomes indispensable. As demonstrated, both native chemical ligation (NCL)^[^ ^39^ ^]^ and, in this study, diselenide–selenoester ligation (DSL) provide highly efficient access to long glycopeptides, even when O‐GalNAc occupies a C‐terminal position of the N‐terminal segment. Moreover, the subsequent deselenization process is so efficient that all steps can be performed by using unpurified crude products. However, it should be noted that there are PTS domains in MUC5AC, which are not accessible to NCL or DSL because all amino acids except proline are glycosylated. In such cases, ligation auxiliaries^[^ ^61^, ^62^ ^]^ must be used, and the results will be reported in due course.

## Conclusion

In conclusion, we have developed a method for the DMF‐free, fast, efficient, and glyco‐economic (0.5 excess equivalents of Fmoc‐Ser/Thr(αAc_3_GalNAc)‐OH) solid‐phase synthesis of mucin peptides, characterized by a hitherto unprecedented degree of GalNAcylation. The method relies on the use of dioxolane (DOL) in all synthetic steps, with the inclusion of the nonionic detergent Tween‐20 during Fmoc removal and coupling. A limit was reached with the synthesis of 26 aa long peptides containing 18 GalNAc residues in three clusters. Longer glycopeptides were synthesized utilizing a convergent approach, which involved the smooth conversion of densely GalNAcylated peptide hydrazides to selenoesters, ligation to massively GalNAcylated selenocysteinyl‐peptides, and selective deselenization in the presence of terminal cysteine residues. CD and PELDOR measurements revealed:

i) Nonglycosylated MUC5AC tandem repeats assume a random coil conformation;
ii) A polyproline type II (PPII) helix conformation is stabilized with progressive α‐*O*‐GalNAcylation, accompanied by a significant increase in the mean end‐to‐end distances;
iii) GalNAcylation at threonine but not at serine induces a polyproline type II helix conformation;
iv) Detection of a PPII helix conformation by CD is not a sufficient indicator for stiffening;
v) A 67% occupancy of potential MUC5AC glycosylation sites (with α‐*O*‐linked GalNAc) is required for stiffening, that is, avoidance of small end‐to‐end distances; and
vi) GalNAcylation of each of the Ser/Thr residues within the APTTSTTS MUC5AC octad repeat induces a helical rise of approx. 2.5 Å per amino acid.

Consequently, it can be concluded that glycosylation with oligosaccharides is not a prerequisite for stiffening the MUC5AC backbone. In future research, we will investigate whether glycosylation with sterically more demanding oligosaccharides may be able to induce stiffening despite a lower degree of glycosylation.

## Supporting Information

The authors have cited additional references within the Supporting Information.^[^ ^63^ ^]^

## Conflict of Interests

The authors declare no conflict of interest.

## Supporting information



Supporting Information

## Data Availability

The data that support the findings of this study are available in the Supporting Information of this article.
