# Supplementary material for: Impact of Glycoclustering on Stiffening of MUC5AC Peptides Revealed by High‐Efficiency Synthesis
Source: Angew Chem Int Ed Engl. 2025 Sep 13;64(44):e202508278. doi: 10.1002/anie.202508278 (PMC12559463; doi:10.1002/anie.202508278)
Supplement: Supplementary file 1 — Supporting Information [file ANIE-64-e202508278-s001.pdf]

Impact of Glycoclustering on Stiffening of MUC5AC Peptides Revealed by High-Efficiency Synthesis

Arseniy Galashov[a], Elisabetta di Gregorio[b], Polina Ponomareva[c], Marc Safferthal[d], Ekaterina Kazakova[a], Leïla Bechtella[d], Kevin Pagel[d], Marina Pigaleva[c], Benesh Joseph[b], Oliver Seitz*[a]

[a] A. Galashov, E. Kazakova, Prof. Dr. O. Seitz
Institut für Chemie
Humboldt-Universität zu Berlin
Brook-Taylor-Straße 2, 12489 Berlin (Germany)
E-mail: oliver.seitz@chemie.hu-berlin.de

[b] Elisabetta di Gregorio, Prof. Dr. B. Joseph
Institut für Experimentalphysik
Freie Universität Berlin
Arnimallee 14, 14195 Berlin (Germany)

[c] Dr. M. Pigaleva, P. Ponomareva
Institut für Chemie und Biochemie
Freie Universität Berlin
Arnimallee 22, 14195 Berlin (Germany)

[d] M. Safferthal, Dr. Leïla Bechtella, Prof. Dr. K. Pagel
Institut für Chemie und Biochemie
Freie Universität Berlin
Altensteinstraße 23A, 14195 Berlin (Germany)

Table of Contents

1. General information 4

2. Synthesis of peptide **1** using DMF 6

2.1 SPPS according to entry 1 of table S1 6

2.2 SPPS according to entries 2 and 3 of table S1 7

2.3 SPPS according to entry 7 of table S1 8

3. SPPS using DOL 9

3.1 SPPS according to entries 4 and 5 of table S1 9

3.2 SPPS according to entry 6 of table S1 10

3.3 Peptides **1-10** 10

Ac-C-(APTTSTTS)$_5$-C-NH$_2$ (**1**) 11

Ac-C-(APT**gT**ST**gT**S)$_5$-C-NH$_2$ (**2**) 11

Ac-C-(AP**gTgT**S**gTgT**S)$_5$-C-NH$_2$ (**3**) 11

UP**gTgTgSgTgTgS** -(AP**gTgTgSgTgTgS**)$_2$-C-NH$_2$ (**4**) 11

Ac-C-(AP**gTgTgSgTgTgS**)$_2$-NH-NH$_2$ (**5**) 11

Ac-C-(APTTSTTS)$_3$-C-NH$_2$ (**6**) 11

Ac-C-(APT**gT**ST**gT**S)$_3$-C-NH$_2$ (**7**) 12

Ac-C-(APTT**gS**TT**gS**)$_3$-C-NH$_2$ (**8**) 12

UP**gTgTgSgTgTgS**-C-NH$_2$ (**10**) 13

4. Synthesis of peptide selenoester **11** 14

Ac-C-(AP**gTgTgSgTgTgS**)$_2$-SePh (**11**) 14

5. Synthesis of peptide **12** via one-pot diselenide-selenoester ligation/deselenization 15

5.1 Diselenide-Selenoester Ligation 15

5.2 Selective deselenization 15

Ac-C-(AP**gTgTgSgTgTgS**)$_5$-C-NH$_2$ (**12**) 15

6. Synthesis of glycopeptide **13** via one-pot selenoesterification/ diselenide-selenoester ligation/deselenization (crude ligation) 16

6.1 Ac-C-(AP**gTgTgSgTgTgS**)$_3$-C-NH$_2$ (**13**) 17

7. CD measurements 18

7.1 CD spectra of GalNAc at varying concentrations 18

7.2 CD spectra of peptides before correction for GalNAc 19

8. Spin labeling of peptides, PELDOR data and CW-EPR data 20

8.1 Spin labeling and HPLC analyses 20

8.2 Primary 4-pulse PELDOR data for peptides **1’**, **2’**, **3’**. 21

8.3 Continuous wave EPR 22

9. Mass spectrometry data 24

9.1 ESI-MS spectra of peptides **4, 5, 11** 24
9.2 ESI-HRMS spectra of peptides **1-3, 12** 25
9.3 ESI-HRMS spectra of peptides **6-9, 13** 26
10. Chemical structure of detergents Tween-20 and Triton X-100 27
11. References 28

1. General information

Commercially available compounds were used without further purification. Tentagel R RAM resin was bought from Rapp Polymere GmbH (Tübingen, Germany). Tentagel R NH_2-NH-TRT resin was prepared according to the published procedure.[1] Fmoc-Thr/Ser(αAc_3GalNAc)-OH was prepared according to the published procedure.[1]

Fmoc-SPPS was performed by using a Biotage Initiator+ Alstra synthesizer (Uppsala, Sweden).

Preparative HPLC purifications were carried out by using an Agilent 1100 Series system equipped with a Nucleodur C18 Gravity column (250 mm x 21 mm, 5 µm) from Macherey-Nagel GmbH & Co. KG (Düren, Germany) with a binary mixture of A (0.1 % TFA, 1 % ACN, 98.9 % H_2O) and B (0.1 % TFA, 1 % H_2O, 98.9 % ACN) as a mobile phase (flow = 15 mL/min) in a linear gradient as described.

HPLC-MS measurements were performed by using an Agilent 1290 Infinity II LC System, equipped with a Poroshell 120 EC-C18 column (2.1 x 50 mm, 1.9 µm; heater set on 50 °C) with a binary mixture of A (0.1 % TFA, 1 % ACN, 98.9 % H_2O) and B (0.1 % TFA, 1 % H_2O, 98.9 % ACN) as a mobile phase (flow = 0.5 mL/min) in a linear gradient as described.

High-Resolution Mass Spectra were recorded in positive ion mode on a Bruker (Bremen, Germany) timsTOF Pro equipped with a custom-built nano-ESI source.[2] Mass calibration was performed using the Agilent ESI-L low concentration tuning mix.

Pulsed electron-electron double resonance (PELDOR) spectroscopy: The spin concentration was quantified using a Bruker EMXnano benchtop spectrometer operating at X-band frequency (9.4 GHz). The spectra were recorded in a 25 µL micropipette (BRAND, Germany) with a 0.64 mm diameter using 100 kHz modulation frequency, 0.15 mT modulation amplitude, 0.6 mW microwave power, 5.12 ms time constant, and 22.5 ms conversion time at room temperature. Samples containing 20% deuterated glycerol (v/v) were then transferred to 1.6 mm quartz EPR tubes and rapidly frozen in liquid nitrogen and stored at -80 °C. PELDOR measurements were performed using a Bruker ELEXSYS E580 Q-band (33.7 GHz) pulsed ESR spectrometer equipped with a SpinJet AWG (Bruker) unit. The system I equipped with a 50 W solid-state amplifier, a continuous-flow helium cryostat, and a temperature control system (Oxford Instruments). Measurements were carried out at 50K using 4-pulse and/or forward 5-pulse DEER sequences with a 16-step phase cycling (reference). In both the cases, the pump and observer pulses were set 80 MHz apart, with the former positioned at the maximum of the echo-detected field swept nitroxide spectrum.

The dead-time free 4-pulse sequence

$$\frac{\pi}{2}_{obs} - \tau_1 - \pi_{obs} - t - \pi_{pump} - (\tau_1 + \tau_2 - t) - \pi_{obs} - \tau_2 - \text{Echo}$$

consisted of a 38 ns Gaussian pump pulse with a full width at half maximum (FWHM) of 16.1 ns; and 48 ns Gaussian observer pulses (FWHM of 20.4 ns).

The 5-pulse sequence

$$\frac{\pi}{2}_{obs} - (\frac{\tau}{2} - t_0) - \pi_{pump} - t_0 - \pi_{obs} - t' - \pi_{pump} - (\tau - t' + \delta) - \pi_{obs} - (\tau_2 + \delta) - \text{Echo}$$

employed 48 ns Gaussian observer pulses, Gaussian pump pulses with standing pump pulse set to 30 ns, and a 48 ns moving pump pulse. Nuclear modulation was averaged as for the 4-

pulse PELDOR (16 ns shift in 8 steps) with a corresponding shift of the standing pump pulse. The data analysis was performed using the DeerLab[3] program.

Circular Dichroism (CD) spectroscopy measurements were performed on a DSM 20 circular dichroism spectrometer (Olis, USA). A 150 µL aliquot of the peptide solution in buffer (25 mM Tris-HCl pH 6.9, 150 mM NaCl) was placed in a quartz flat cell with a 1 mm light path and placed inside a custom-made holder for flat, low-volume cells. 100 data points were measured in each of the three scans, which were averaged for each spectrum. The spectra were recorded within a range of 190–260 nm at a scan rate of 2 nm per minute. The spectrum of the buffer solution was measured before the peptide solution and subtracted. For comparison of different peptides, the spectra were measured at a consistent concentration of 50 µM for each solution.

To correct the CD-spectra of glycopeptides, pure GalNAc solutions were measured, with concentrations of 300, 500, 600, 900, 1000 and 1500 µM. Spectral contributions of pure GalNAc were subtracted based on the number of glycosylated groups in each peptide: peptides **7**, **8** were corrected with 300 µM of GalNAc, **9** was corrected with 600 µM of GalNAc, **13** was corrected with 900 µM of GalNAc, **2** was corrected with 500 µM of GalNAc, **3** was corrected with 1000 µM of GalNAc, **12** was corrected with 1500 µM of GalNAc. All spectra were baseline corrected at 245 nm.

2. Synthesis of peptide **1** using DMF

Automated synthesis was performed at 40 μmol scale on Tentagel R Rink Amide resin (0.20 mmol/g) by using a Biotage Initiator+ Alstra synthesizer (Uppsala, Sweden).

Table S1. Synthesis of 42 amino acid MUC5AC peptide **1** (C(APTTSTTS)$_5$C).[a]

	Conditions[a]	Solvent	Pseudo-proline[b]	Tween-20	Crude purity[c]
1	a1-b1-c1	DMF	-	-	N.d.
2	a2-b2-c1	DMF	-	-	N.d.
3	a2-b2-c1	DMF	+	-	4%
4	a3-b3-c2	DOL	-	-	39%
5	a3-b3-c2	DOL	+	-	50%
6	a4-b4-c2	DOL	-	+	63%
7	a5-b5-c1	DMF	-	+	3%

[a] Fmoc removal: a1) 20% piperidine in DMF, 1x1 min, 90°C; a2) 20% piperidine in DMF, 2x5 min, RT; a3) 20% pyrrolidine in DOL, 2x5 min, RT; a4) 20% pyrrolidine in DOL/1% Tween-20, 2x5 min, RT; a5) 20% piperidine in DMF/1% Tween-20, 2x5 min, RT. Coupling: b1) 5 eq. AA/DIC/Oxyma in DMF, 1 min, 90°C; b2) 5 eq. AA/DIC/Oxyma in DMF, 5 min, 75°C; b3) 5 eq. AA/DIC/Oxyma in DOL, 5 min, RT; b4) 5 eq. AA/DIC/Oxyma in DOL/1% Tween-20, 5 min, RT; b5) 5 eq. AA/DIC/Oxyma in DMF/1% Tween-20, 5 min, 75°C. Capping: c1) Ac_2O/DIPEA (20%/10%) in DMF, 5 min, RT; c2) Ac_2O/DIPEA (20%/10%) in DOL, 5 min, RT. [b] Pseudoproline was inserted in the C-terminal Tandem Repeat (APTTST^{psi}TS) [c] Purity of crudes based on HPLC analysis at 210 nm. N.d. – not determined.

2.1 SPPS according to entry 1 of table S1

Fmoc-removal: The resin was treated with deprotection solution (20% Piperidine in DMF) for 1x1 min at 90°C. The resin was washed with 3x2 mL of DMF, each washing was performed for 1 minute.

Coupling: Fmoc-protected amino acids (5 eq.) were transferred to the resin, followed by DIC and Oxyma (5 eq. each). The final concentration of amino acid was 167 mM. Reaction temperature was elevated to 90°C (coupling time = 2 min). The resin was washed with 2x2 mL of DMF, each washing was performed for 1 minute.

Capping: The resin was treated with DMF:Ac_2O:DiPEA (70:20:10, v/v/v) for 5 min at RT. The resin was washed with 2x2 mL of DMF, each washing was performed for 1 minute.

TFA cleavage: Prior to TFA cleavage the Fmoc group was removed as described, and final capping procedure was performed as described. The resin was washed with CH_2Cl_2 and dried under vacuum. Then 5 mL of a mixture of TFA:TIS:H_2O:EDT (94:2:2:2, v/v/v) was added to the resin. After 2 h the cleavage cocktail was collected by filtration, the resin was washed once with 5 mL of TFA and the combined filtrates were concentrated under argon flow.

Peptide work-up: To the remaining residue cold Et_2O (9-fold volume) was added and the suspension was centrifuged (4200 rpm, 5 min). Afterwards the ether phase was decanted. The peptide precipitate was dissolved in the mixture of 6M Gd-Cl/ACN (9:1).

2.2 SPPS according to entries 2 and 3 of table S1

Fmoc-removal: The resin was treated with deprotection solution (20% Piperidine in DMF) for 2x5 min at RT. The resin was washed with 3x2 mL of DMF, each washing was performed for 1 minute.

Coupling: Fmoc-protected amino acids (5 eq.) were transferred to the resin, followed by DIC and Oxyma (5 eq. each). The final concentration of amino acid was 167 mM. Reaction temperature was elevated to 75°C (coupling time = 5 min). The resin was washed with 2x2 mL of DMF, each washing was performed for 1 minute. The method described in entry 3 of table S1 involved the introduction of pseudoproline into the C-terminal Tandem Repeat in the third coupling (APTT**ST**^{psi}TS).

Capping: The resin was treated with DMF:Ac_2O:DiPEA (70:20:10, v/v/v) for 5 min at RT. The resin was washed with 2x2 mL of DMF, each washing was performed for 1 minute.

TFA cleavage: Prior to TFA cleavage the Fmoc group was removed as described, and final capping procedure was performed as described. The resin was washed with CH_2Cl_2 and dried under vacuum. Then 5 mL of a mixture of TFA:TIS:H_2O:EDT (94:2:2:2, v/v/v) was added to the resin. After 2 h the cleavage cocktail was collected by filtration, the resin was washed once with 5 mL of TFA and the combined filtrates were concentrated under argon flow to 5 mL.

Peptide work-up: To the remaining residue cold Et_2O (9-fold volume) was added and the suspension was centrifuged (4200 rpm, 5 min). Afterwards the ether phase was decanted. The peptide precipitate was dissolved in the mixture of 6M Gd-Cl/ACN (9:1).

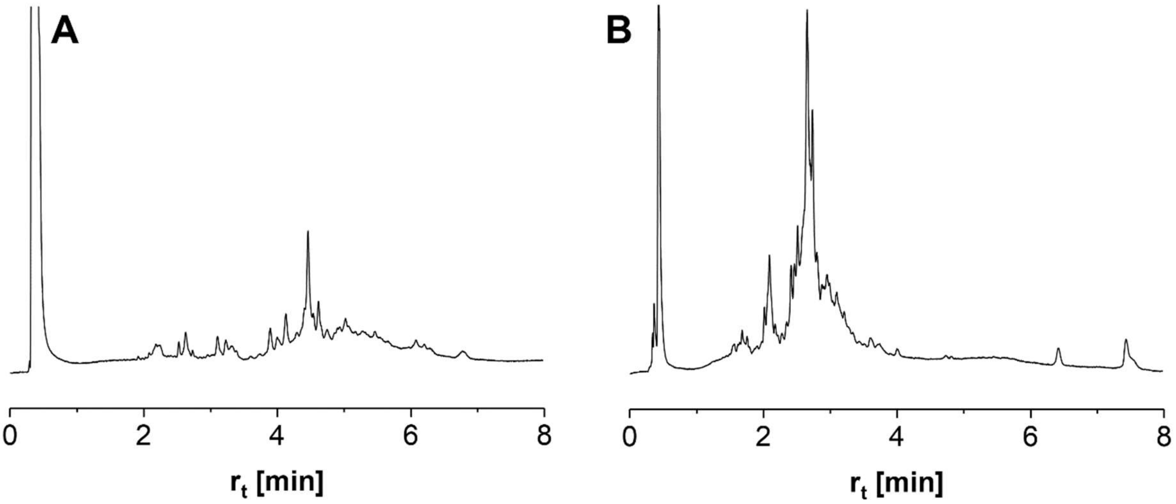

Figure S1. HPLC analysis of crude material obtained in attempts to synthesize peptide **1** by using methods described in A) entry 1 of table S1 and B) entry 2 of table S1. Conditions: Gradients for A): 03-30% ACN in Water + 0.1% TFA; for B): 03-60% ACN in Water + 0.1% TFA. λ = 210 nm. MS analysis did not show peaks with m/z-values expected for peptide **1**.

2.3 SPPS according to entry 7 of table S1

Fmoc-removal: The resin was treated with deprotection solution (20% Piperidine in DMF + 1% Tween-20 (v/v)) for 2x5 min. The resin was washed with 3x2 mL of DMF, each washing was performed for 1 minute.

Coupling: Fmoc-protected amino acids (5 eq.) were transferred to the resin, followed by DIC and Oxyma (5 eq. each). The final concentration of amino acid was 167 mM, final reaction mixture contained 1% Tween-20 (v/v). Reaction temperature was elevated to 75°C (coupling time = 5 min). The resin was washed with 2x2 mL of DMF, each washing was performed for 1 minute.

Capping: The resin was treated with DMF:Ac_2O:DiPEA (70:20:10, v/v/v) for 5 min at RT. The resin was washed with 2x2 mL of DMF, each washing was performed for 1 minute.

TFA cleavage: Prior to TFA cleavage the Fmoc group was removed as described, and final capping procedure was performed as described. The resin was washed with CH_2Cl_2 and dried under vacuum. Then 5 mL of a mixture of TFA:TIS:H_2O:EDT (94:2:2:2, v/v/v) was added to the resin. After 2 h the cleavage cocktail was collected by filtration, the resin was washed once with 5 mL of TFA and the combined filtrates were concentrated under argon flow to 5 mL.

Peptide work-up: To the remaining residue cold Et_2O (9-fold volume) was added and the suspension was centrifuged (4200 rpm, 5 min). Afterwards the ether phase was decanted. The peptide precipitate was dissolved in the mixture of 6M Gd-Cl/ACN (9:1).

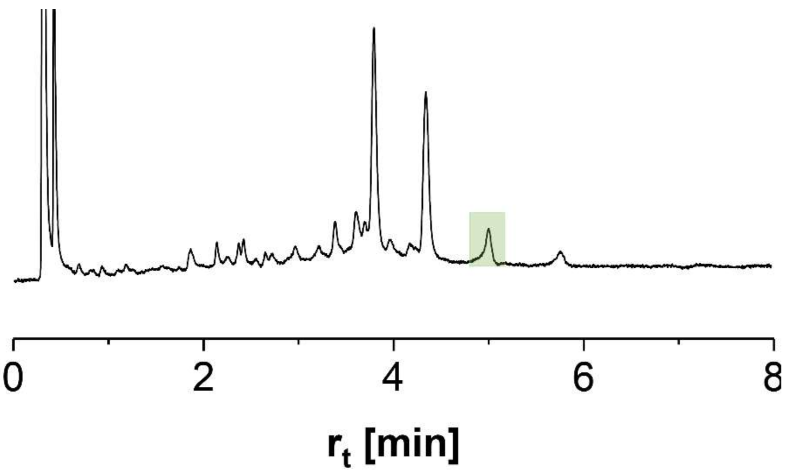

Crude purity: 3%

Figure S2. HPLC analysis of crude peptide **1** obtained by using the method described in entry 7 of table S1. Conditions: Gradient: 05-20% ACN in Water + 0.1% TFA. λ = 210 nm. Green area marks target compound.

3. SPPS using DOL

Automated synthesis was performed at 40 µmol scale on Tentagel R Rink Amide resin (0.20 mmol/g) by using a Biotage Initiator+ Alstra synthesizer (Uppsala, Sweden).

3.1 SPPS according to entries 4 and 5 of table S1

Fmoc-removal: The resin was treated with deprotection solution (20% Pyrrolidine in DOL) for 2x5 min. The resin was washed with 3x2 mL of DOL, each washing was performed for 1 minute.

Coupling: Fmoc-protected amino acids (5 eq.) were transferred to the resin, followed by DIC and Oxyma (5 eq. each). The final concentration of amino acid was 167 mM. Reaction was performed at RT (coupling time = 5 min). The resin was washed with 2x2 mL of DOL, each washing was performed for 1 minute. The method described in entry 5 of table S1 involved the introduction of pseudoproline into the C-terminal Tandem Repeat in the third coupling (APTT**ST**^{psi}TS).

Capping: The resin was treated with DOL:Ac_2O:DiPEA (70:20:10, v/v/v) for 5 min at RT. The resin was washed with 2x2 mL of DOL, each washing was performed for 1 minute.

TFA cleavage: Prior to TFA cleavage the Fmoc group was removed as described, and final capping procedure was performed as described. The resin was washed with CH_2Cl_2 and dried under vacuum. Then 5 mL of a mixture of TFA:TIS:H_2O:EDT (94:2:2:2, v/v/v) was added to the resin. After 2 h the cleavage cocktail was collected by filtration, the resin was washed once with 5 mL of TFA and the combined filtrates were concentrated under argon flow to 5 mL.

Peptide work-up: To the remaining residue cold Et_2O (9-fold volume) was added and the suspension was centrifuged (4200 rpm, 5 min). Afterwards the ether phase was decanted. The peptide precipitate was dissolved in the mixture of 6M Gd-Cl/ACN (9:1).

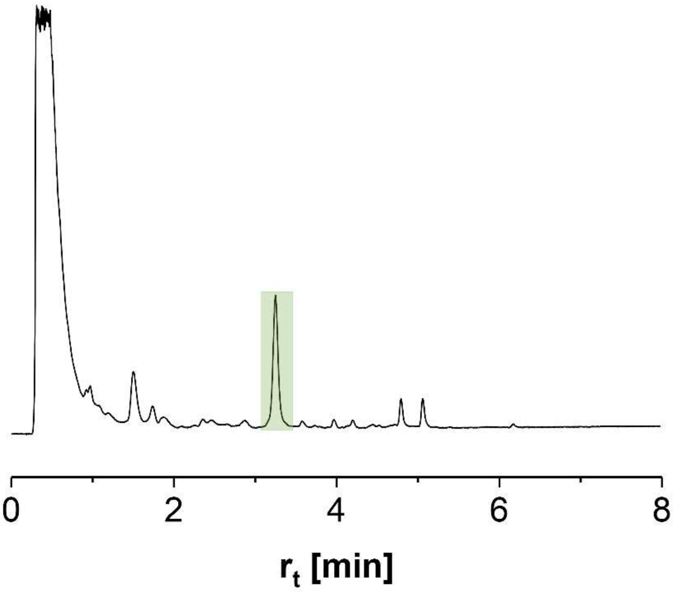

Crude purity: 39%

Figure S3. HPLC analysis of crude peptide **1** obtained by using the method described in entry 4 of table S1. Conditions: Gradient: 10-25% ACN in Water + 0.1% TFA. λ = 210 nm. Green area marks target compound.

3.2 SPPS according to entry 6 of table S1

Fmoc-removal: The resin was treated with deprotection solution (20% Pyrrolidine in DOL + 1% Tween-20 (v/v)) for 2x5 min. The resin was washed with 3x2 mL of DOL, each washing was performed for 1 minute.

Coupling: Fmoc-protected amino acids (5 eq.) were transferred to the resin, followed by DIC and Oxyma (5 eq. each). The final concentration of amino acid was 167 mM. Reaction was performed at RT, final reaction mixture contained 1% Tween-20 (v/v) (coupling time = 5 min). The resin was washed with 2x2 mL of DOL, each washing was performed for 1 minute.

Capping: The resin was treated with DOL:Ac_2O:DiPEA (70:20:10, v/v/v) for 5 min at RT. The resin was washed with 2x2 mL of DOL, each washing was performed for 1 minute.

TFA cleavage: Prior to TFA cleavage the Fmoc group was removed as described, and final capping procedure was performed as described. The resin was washed with CH_2Cl_2 and dried under vacuum. Then 5 mL of a mixture of TFA:TIS:H_2O:EDT (94:2:2:2, v/v/v) was added to the resin. After 2 h the cleavage cocktail was collected by filtration, the resin was washed once with 5 mL of TFA and the combined filtrates were concentrated under argon flow to 5 mL.

Peptide work-up: To the remaining residue cold Et_2O (9-fold volume) was added and the suspension was centrifuged (4200 rpm, 5 min). Afterwards the ether phase was decanted. The peptide precipitate was dissolved in the mixture of 6M Gd-Cl/ACN (9:1).

3.3 Peptides **1-10**

Peptide **5** was prepared with Tentagel R NH_2-NH-TRT resin. Syntheses were performed according to the method described in 3.2 with the following adjustments:

Coupling of usual amino acids: Fmoc-protected amino acids (6 eq.) were transferred to the resin, followed by DIC and Oxyma (6 eq. each). Coupling mixture contained 120 mM of amino acid and 1% Tween-20 (v/v). Coupling time was extended to 10 min. The resin was washed with 1x1 ml of DMF and 2x2 mL of DOL, each washing was performed for 1 minute.

Coupling of glycoamino acids: Fmoc-protected amino acids (1.5 eq.) were transferred to the resin, followed by DIC and Oxyma (1.5 eq. each). Coupling mixture contained 60 mM of amino acid and 1% Tween-20 (v/v). Coupling time was 10 min. The resin was washed with 1x1 ml of DMF and 2x2 mL of DOL, each washing was performed for 1 minute.

Capping: The resin was treated with DOL:Ac_2O:DiPEA (70:20:10, v/v/v) for 5 min at RT. The resin was washed with 3x2 mL of DOL, each washing was performed for 1 minute.

Removal of O-acetyl groups on resin (for glycopeptides **2**-**5**, **7**-**10**): 1 mL of DMF-$N_2H_{4\ (aq.)}$ (1:1, v/v) was added to the resin. After 30 minutes, mixture was removed and resin was washed with 5xL ml of DMF, 5x1 mL of CH_2Cl_2, and 2x1 mL of Et_2O.

TFA cleavage: Prior to TFA cleavage the Fmoc group was removed as described, final capping procedure was performed as described and O-acetyl groups were removed as described. Then 5 mL of a mixture of TFA:TIS:H_2O:EDT (94:2:2:2, v/v/v) was added to the resin. After 2 h the cleavage cocktail was collected by filtration, the resin was washed once with 5 mL of TFA and the combined filtrates were concentrated under argon flow.

Peptide work-up: To the remaining residue cold Et_2O (9-fold volume) was added and the suspension was centrifuged (4200 rpm, 5 min). Afterwards the ether phase was decanted. The precipitate from glycopeptides was dissolved in Mili-Q Water. Non-glycosylated peptides were dissolved in a mixture 6M Gd-Cl/ACN (9:1).

Ac-C-(APTTSTTS)$_5$-C-NH$_2$ (**1**)

Preparative HPLC 10-18% B in 40 min. Yield: 28.2 mg (7.1 µmol), 18%. UPLC: t_R = 5.66 min (05-20% B in 10 min); ESI-HRMS (pos. mode): m/z = 1333.2689 ($C_{158}H_{267}N_{43}O_{73}S_2$ $(M+2H)^{2+}$, calcd.: 1333.2674).

Ac-C-(APT**gT**ST**gT**S)$_5$-C-NH$_2$ (**2**)

Preparative HPLC 05-12% B in 40 min. Yield: 38.6 mg (6.4 µmol), 16%. UPLC: t_R = 4.90 min (05-15% B in 10 min); ESI-HRMS (pos. mode): m/z = 1333.2689 ($C_{238}H_{398}N_{53}O_{123}S_2$ $(M+3H)^{3+}$, calcd.: 1333.2674), 2010.1954 ($C_{238}H_{399}N_{53}O_{123}S_2$ $(M+4H)^{4+}$, calcd.: 2010.1986).

Ac-C-(AP**gTgT**S**gTgT**S)$_5$-C-NH$_2$ (**3**)

Preparative HPLC 04-09% B in 40 min. Yield: 7.2 mg (0.9 µmol), 2%. UPLC: t_R = 4.51 min (00-10% B in 10 min); ESI-HRMS (pos. mode): m/z = 2015.6003 ($C_{318}H_{529}N_{63}O_{173}S_2$ $(M+4H)^{4+}$, calcd.: 2015.5994).

UP**gTgTgSgTgTgS** -(AP**gTgTgSgTgTgS**)$_2$-C-NH$_2$ (**4**)

Preparative HPLC 00-10% B in 40 min. Yield: 11.8 mg (1.9 µmol), 5%. UPLC: t_R = 4.86 min (00-10% B in 10 min); ESI-MS (pos. mode): m/z = 1525.4 ($C_{237}H_{396}N_{44}O_{133}SSe$ $(M+4H)^{4+}$, calcd.: 1524.6).

Ac-C-(AP**gTgTgSgTgTgS**)$_2$-NH-NH$_2$ (**5**)

Preparative HPLC 00-10% B in 40 min. Yield: 41.7 mg (10.7 µmol), 27%. UPLC: t_R = 5.46 min (02-12% B in 10 min); ESI-MS (pos. mode): m/z = 1370.3 ($C_{161}H_{270}N_{31}O_{90}S$ $(M+3H)^{3+}$, calcd.: 1369.9), 1027.9 ($C_{161}H_{271}N_{31}O_{90}S$ $(M+4H)^{4+}$, calcd.: 1027.7).

Ac-C-(APTTSTTS)$_3$-C-NH$_2$ (**6**)

Preparative HPLC 05-20% B in 40 min. Yield: 17.5 mg (7.0 µmol), 18%. UPLC: t_R = 5.02 min (05-20% B in 10 min); ESI-HRMS (pos. mode): m/z = 1253.5524 ($C_{98}H_{167}N_{27}O_{45}S_2$ $(M+2H)^{2+}$, calcd.: 1253.5520).

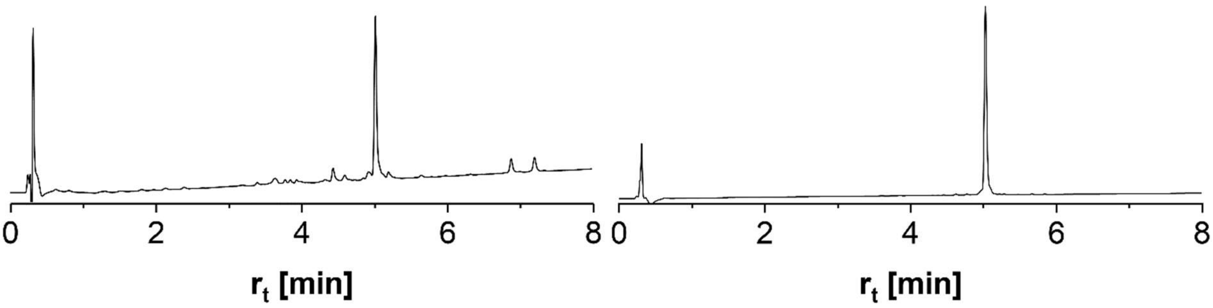

Figure S4. HPLC analysis of peptide **6** before (left) and after (right) HPLC purification. Gradient 05-20% ACN in Water + 0.1% TFA, λ = 210 nm.

Ac-C-(APT**gT**ST**gT**S)$_3$-C-NH$_2$ (**7**)

Preparative HPLC 05-15% B in 40 min. Yield: 23.8 mg (6.4 µmol), 16%. UPLC: t_R = 4.46 min (05-15% B in 10 min); ESI-HRMS (pos. mode): m/z = 1242.1957 ($C_{147}H_{247}N_{32}O_{75}S_2$ $(M+3H)^{3+}$, calcd.: 1242.1958).

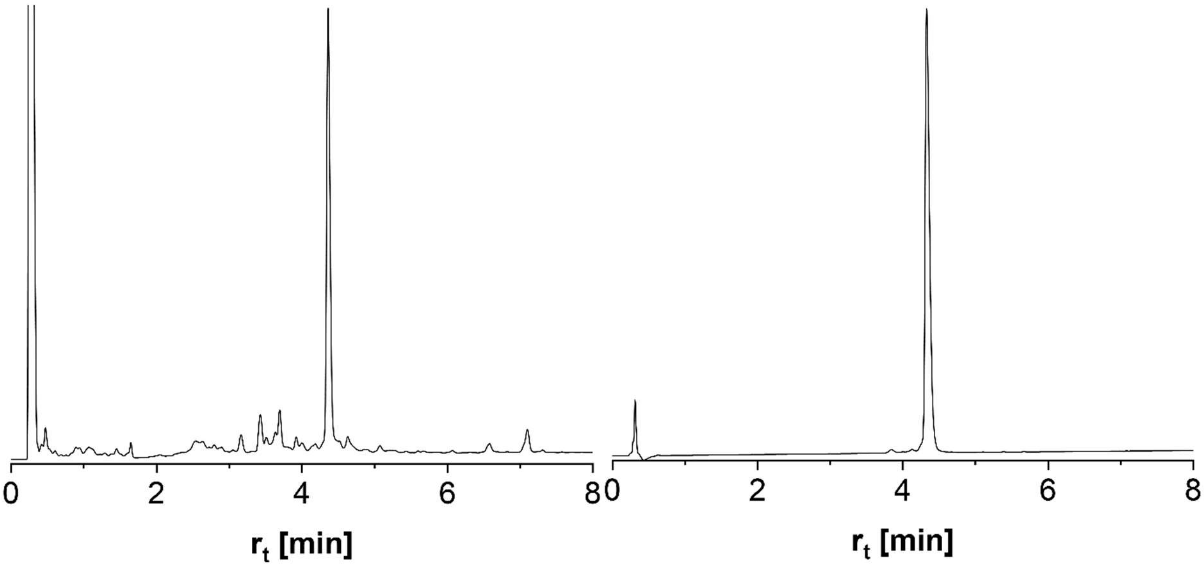

Figure S5. HPLC analysis of peptide **7** before (left) and after (right) HPLC purification. Gradient 05-15% ACN in Water + 0.1% TFA, λ = 210 nm.

Ac-C-(APTT**gS**TT**gS**)$_3$-C-NH$_2$ (**8**)

Preparative HPLC 07-15% B in 40 min. Yield: 13.4 mg (3.6 µmol), 9%. UPLC: t_R = 4.53 min (07-15% B in 10 min); ESI-HRMS (pos. mode): m/z = 2054.8457 ($C_{147}H_{246}N_{32}O_{75}S_2$ $(M+2H)^{2+}$, calcd.: 2054.8466), 1242.1961 ($C_{147}H_{247}N_{32}O_{75}S_2$ $(M+3H)^{3+}$, calcd.: 1242.1958).

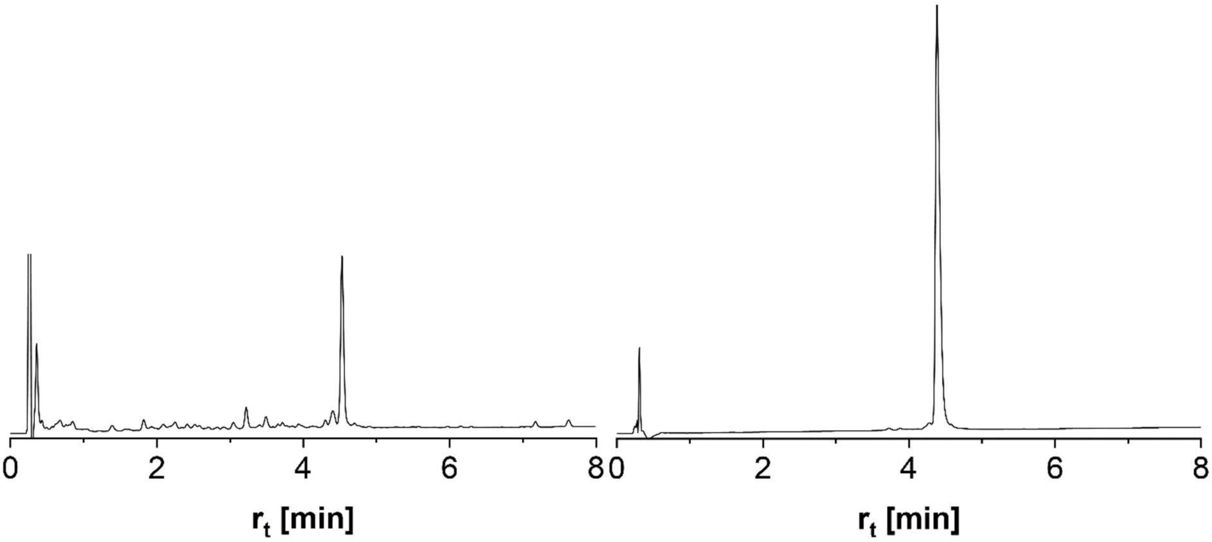

Figure S6. HPLC analysis of peptide **8** before (left) and after (right) HPLC purification. Gradient 07-15% ACN in Water + 0.1% TFA, λ = 210 nm.

Ac-C-(APgTgTSgTgTS)3-C-NH2 (**9**)

Preparative HPLC 02-12% B in 40 min. Yield: 19.3 mg (3.9 µmol), 10%. UPLC: t_R = 5.52 min (02-12% B in 10 min); ESI-HRMS (pos. mode): m/z = 1648.6883 ($C_{194}H_{324}N_{39}O_{105}S_2$ $(M+3H)^{3+}$, calcd.: 1648.6879).

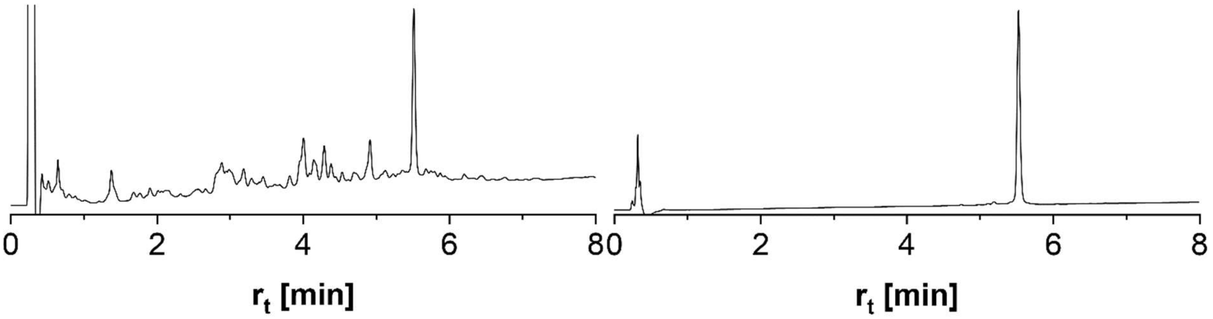

Figure S7. HPLC analysis of peptide **9** before (left) and after (right) HPLC purification. Gradient 02-12% ACN in Water + 0.1% TFA, λ = 210 nm.

UPgTgTgSgTgTgS-C-NH2 (**10**)

The terminal selenocysteine was introduced by means of Fmoc-L-Sec(Xan)-OH. N-terminal capping was omitted. Glycopeptide **10** was not purified.

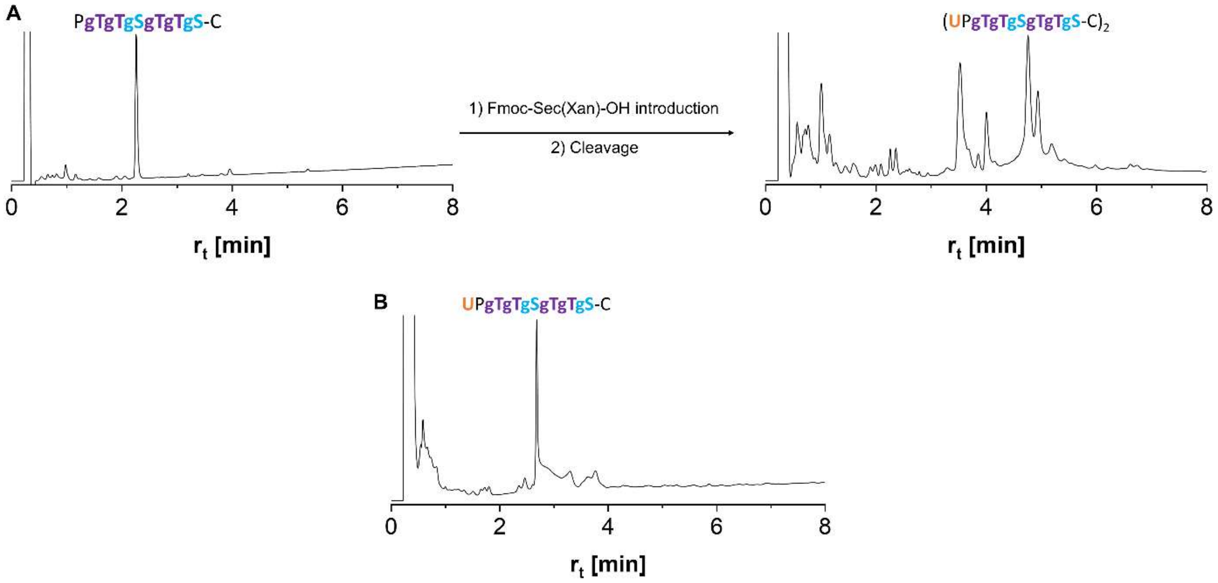

Figure S8. HPLC analysis of A) crudes obtained before (left) and after (right) Fmoc-Sec(Xan)-OH was introduced, and B) crude material treated with TCEP-HCl. Multiple peaks found in the right trace of A indicate the formation of various isoforms containing Se-Se, S-Se and S-S bonds in monomers/dimers/trimers/etc. Gradient 00-10% ACN in Water + 0.1% TFA, λ = 210 nm.

---

4. Synthesis of peptide selenoester **11**

Glycopeptide hydrazide **5** (7.1 mg, 1.73 µmol) was dissolved in 336 µL of buffer (6M Gd-HCl, 200mM Na_2HPO_4, pH = 8.5) to reach 5 mM concentration. TCEP-HCl (19.67 mg, 68.62 µmol) was added to the mixture to reach 200 mM concentration. 15 µL of 1M NaOH were added to adjust pH to 3.0. Ph_2Se_2 (5.6 mg, 17.94 µmol) was added to reach 100 mM concentration. Acetylacetone (2 µL, 19.50 µmol) was added to reach 56 mM concentration. The mixture was vortexed, and put to the shaker at 37°C. After 2 hours, the formation of glycopeptide selenoester **11** was finished and reaction mixture was submitted to purification by preparative HPLC (02-12% B in 40 min).

Ac-C-(AP**gTgTgSgTgTgS**)$_2$-SePh (**11**)

Preparative HPLC 02-12% B in 40 min. Yield: 4.1 mg (1.0 µmol), 58%. UPLC: t_R = 6.43 min (02-12% B in 10 min); ESI-MS (pos. mode): m/z = 1412.2 ($C_{167}H_{272}N_{29}O_{90}SSe$ $(M+3H)^{3+}$, calcd.: 1411.9), 1059.7 ($C_{167}H_{273}N_{29}O_{90}SSe$ $(M+4H)^{4+}$, calcd.: 1059.2).

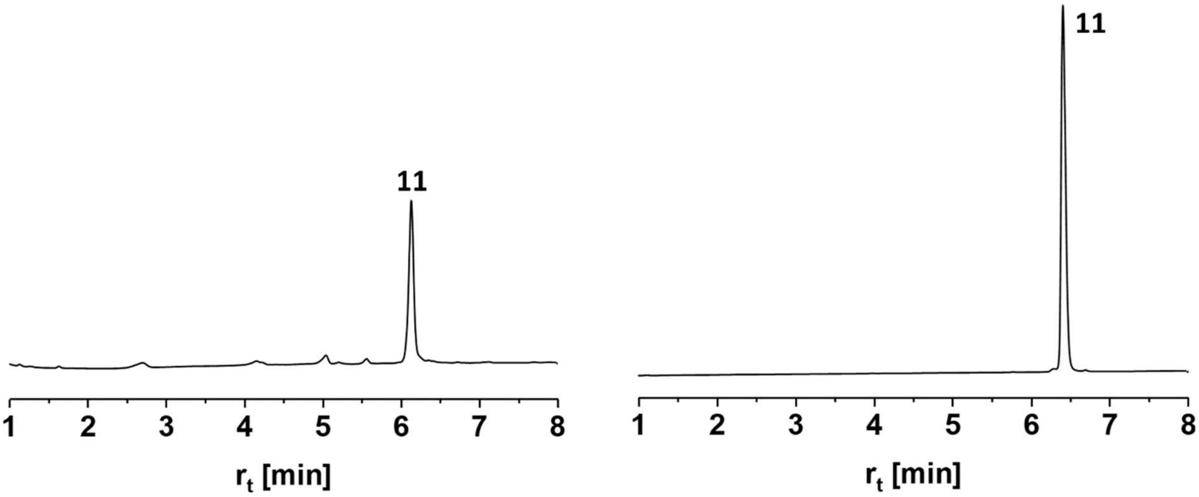

Figure S9. HPLC analysis of peptide selenoester **11** before (left) and after (right) HPLC purification. Gradient 02-12% ACN in Water + 0.1% TFA, λ = 210 nm.

5. Synthesis of peptide **12** via one-pot diselenide-selenoester ligation/deselenization

5.1 Diselenide-Selenoester Ligation

Ac-C-(APgTgTgSgTgTgS)$_2$-UPgTgTgSgTgTgS-(APgTgTgSgTgTgS)$_2$-C-NH$_2$ (**S12**)

Glycopeptide **4** (6.10 mg, 1.00 µmol) was dissolved in 200 µL of buffer (6M Gd-HCl, 200mM Na_2HPO_4, pH = 6.9) to reach 5 mM concentration. The mixture was combined with glycopeptide selenoester **11** (5.00 mg, 1.20 µmol) in a separate tube; concentration of **11** reached 6 mM. After 10 minutes, additional glycopeptide selenoester **11** (4.2 mg, 1.00 µmol) was added. The reaction was allowed to proceed for additional 10 minutes. Afterwards, the mixture was forwarded to one-pot deselenization.

For HPLC analyses, aliquots were withdrawn and reaction was quenched by addition of N_2H_4. A small crystal of TCEP-HCl was added to break Se-S bonds and facilitate analysis.

5.2 Selective deselenization

Hexane (200 µL) was added to the peptide ligation mixture. The mixture was vigorously vortexed, and the upper hexane layer was removed with a pipette. The extraction procedure was repeated twice. The aqueous solution was degassed with argon. 1 mL of aqueous solution of TCEP/DTT (71.66 mg, 250 mM/ 3.86 mg, 25 mM) was prepared, the pH was adjusted to 4.6 with 1M NaOH and the solution was degassed with argon. The crude ligation mixture was treated with equal volume of deselenization solution (200 µL) to give a final concentration of 2.5 mM of peptide ligation product. The reaction was allowed to proceed overnight. Following completion of the reaction, the mixture was submitted to purification by preparative HPLC (02-12% B in 40 min).

Ac-C-(APgTgTgSgTgTgS)$_5$-C-NH$_2$ (**12**)

Preparative HPLC 02-12% B in 40 min. Yield: 2.12 mg (0.21 µmol), 22%. UPLC: t_R = 4.51 min (02-12% B in 10 min); ESI-HRMS (pos. mode): m/z = 2018.8396 ($C_{398}H_{670}N_{73}O_{223}S_2$ $(M+5H)^{5+}$, calcd.: 2018.8398).

6. Synthesis of glycopeptide **13** via one-pot selenoesterification/ diselenide-selenoester ligation/deselenization (crude ligation)

Glycopeptide hydrazide **5** (12.0 mg, 2.8 μmol) was dissolved in 528 μL of buffer (6M Gd-HCl, 200mM Na_2HPO_4, pH = 8.5) to reach 5 mM concentration. TCEP-HCl (26.5 mg, 105.9 μmol) was added to the mixture to reach 200 mM concentration. pH of mixture became 3.1. Ph_2Se_2 (16.5 mg, 29.8 μmol) was added to reach 100 mM concentration. Acetylacetone (3.1 μL, 29.7 nmol) was added to reach 56 mM concentration. The mixture was vortexed, and put to the shaker at 37°C. After 2 hours, the formation of glycopeptide selenoester **11** was finished.

Crude glycopeptide **U**P**gTgTgSgTgTgS**-NH_2 (**10**) (14 mg) was added to the reaction mixture. Afterwards, the pH of the mixture was raised to 6.9 by adding 85 μL of 5M NaOH. After 20 minutes, following completion of the reaction, the mixture was forwarded to the deselenization step. Formation of unproductive selenoester was inhibited in the presence of DPDS-TCEP.

Hexane (400 μL) was added to the peptide ligation mixture. The mixture was vigorously vortexed, and the upper hexane layer was removed with a pipette. The extraction procedure was repeated twice. The aqueous solution was degassed with argon. Solid TCEP-HCl (79 mg) and DTT (4.3 mg) were added to the reaction mixture. The pH was adjusted to 5.0 with 152 μL of 5M NaOH. The reaction was allowed to proceed overnight. Following completion of the reaction, the mixture was submitted to purification by preparative HPLC (02-12% B in 40 min).

6.1 Ac-C-(APgTgTgSgTgTgS)$_3$-C-NH$_2$ (**13**)

Preparative HPLC 02-12% B in 40 min. Yield: 3.1 mg (0.5 µmol), 18%. UPLC: t_R = 4.35 min (02-12% B in 10 min); ESI-HRMS (pos. mode): m/z = 2054.8457 ($C_{242}H_{402}N_{45}O_{135}S_2$ $(M+3H)^{3+}$, calcd.: 2054.8466), 1541.3849 ($C_{242}H_{403}N_{45}O_{135}S_2$ $(M+4H)^{4+}$, calcd.: 1541.3868).

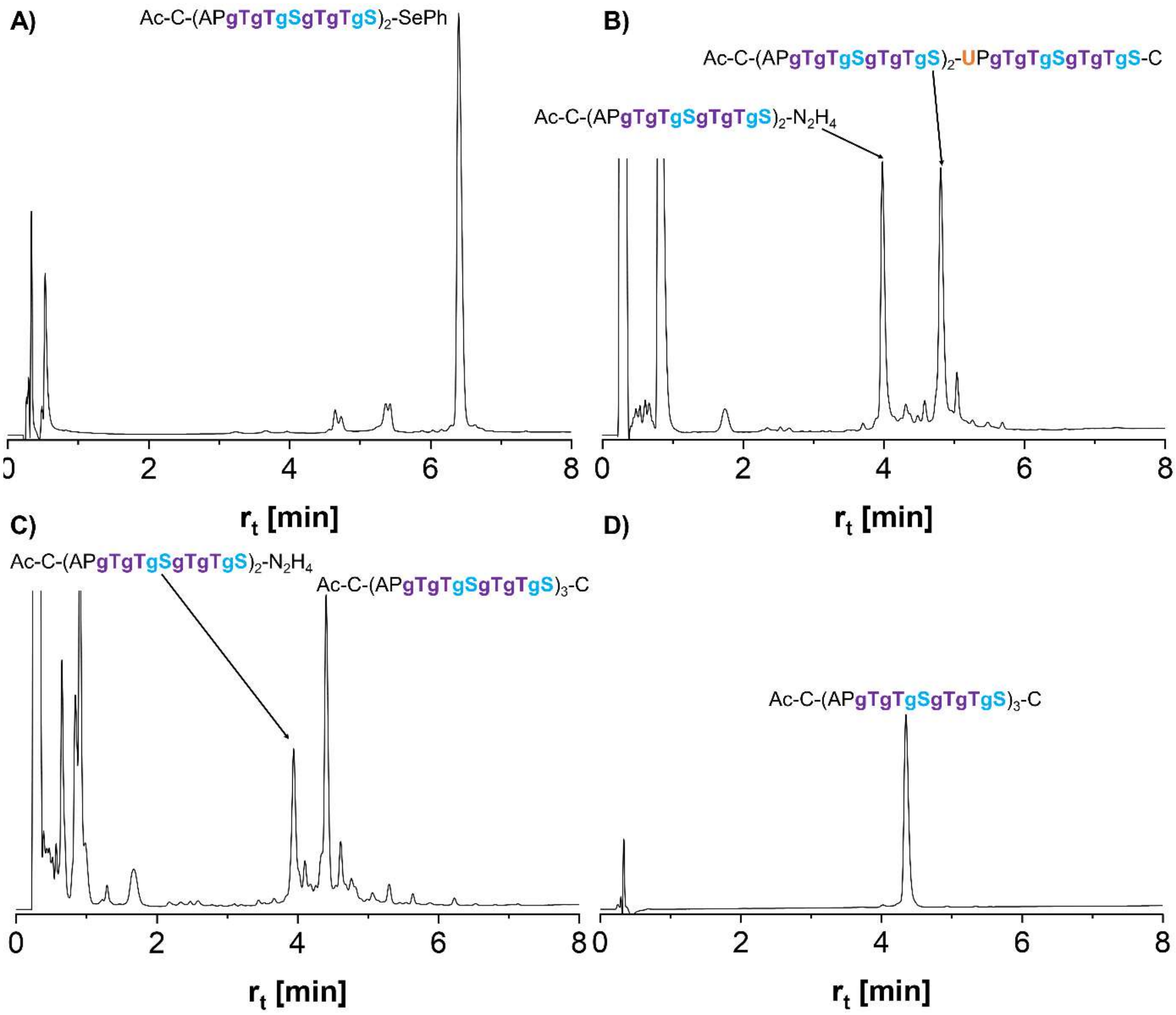

Figure S10. HPLC analysis of A) peptide selenoester **11** prepared to start one-pot reaction cascade, B) reaction mixture after 10 minutes from the addition of crude peptide **10**, C) reaction mixture after deselenization, D) purified peptide **13**. N_2H_4 was added to samples before HPLC analysis, converting peptide selenoester **11** to peptide hydrazide **5**. Gradient 02-12% ACN in Water + 0.1% TFA, λ = 210 nm.

7. CD measurements

7.1 CD spectra of GalNAc at varying concentrations

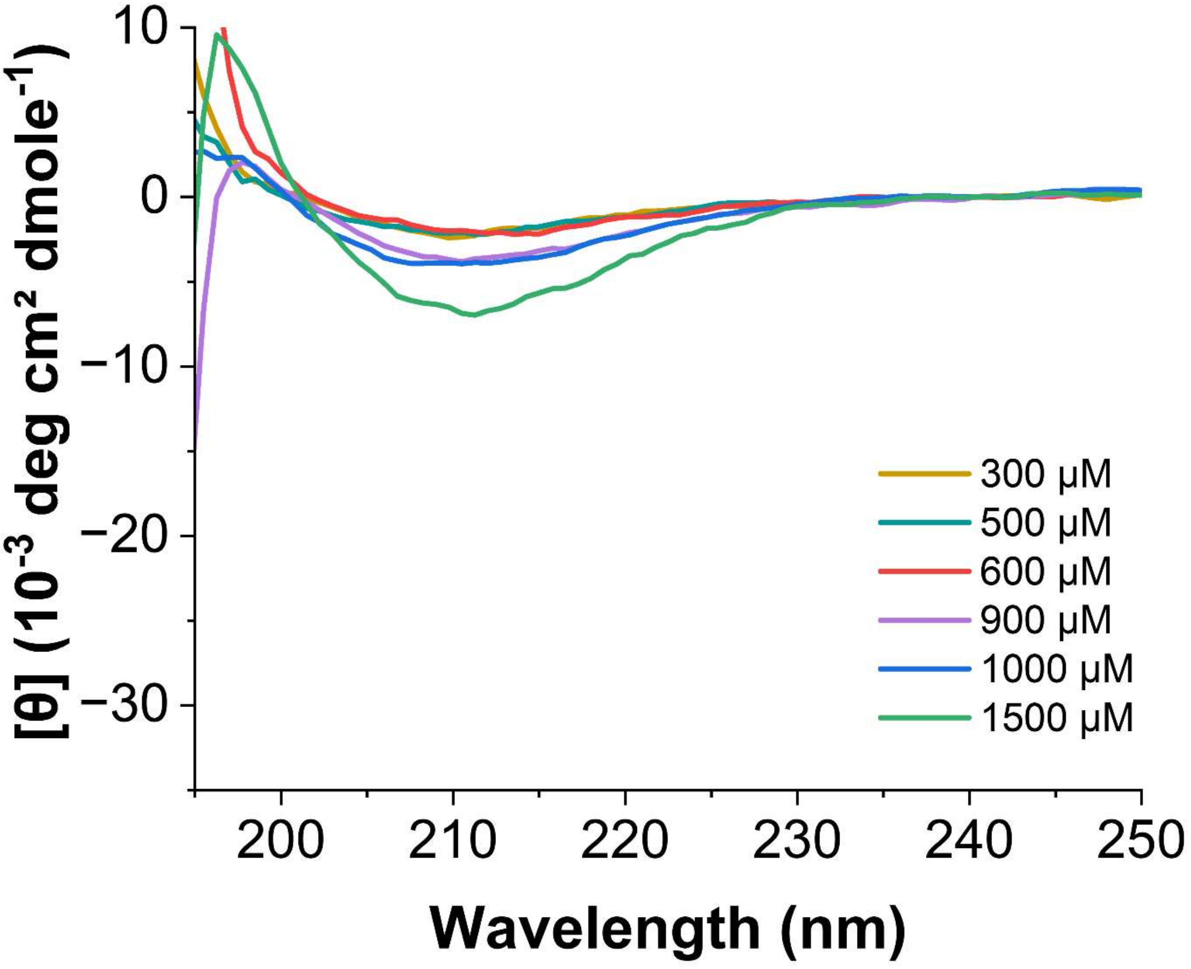

Figure S11. CD spectra of pure GalNAc at 300, 500, 600, 900, 1000 and 1500 μM.

7.2 CD spectra of peptides before correction for GalNAc

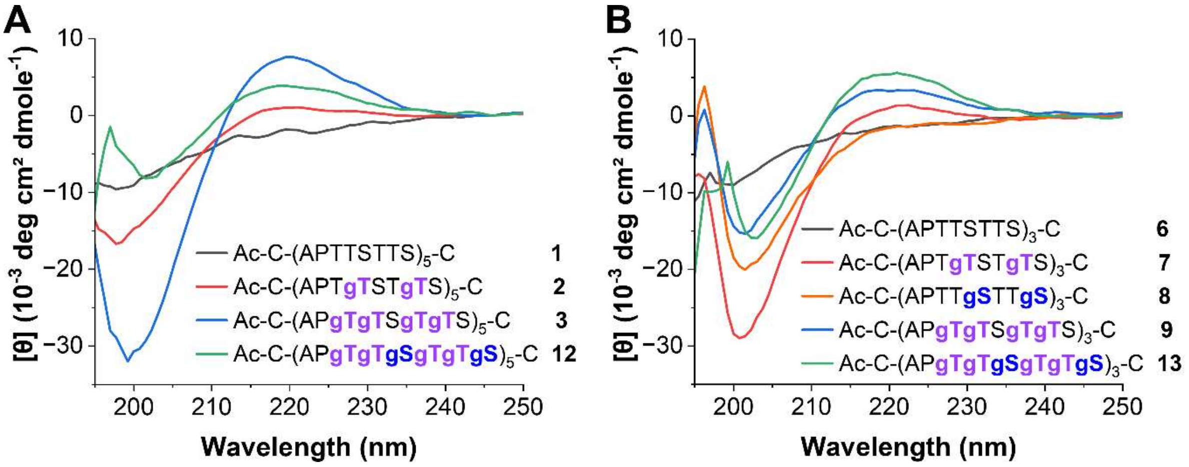

Figure S12. CD spectra of peptides containing A) five tandem repeats (1-3,12) or B) three tandem repeats (6-9, 13).

8. Spin labeling of peptides, PELDOR data and CW-EPR data

8.1 Spin labeling and HPLC analyses

Spin labeling of peptides was performed for 1 h at room temperature with 700 µM final spin label concentrations for 1-oxyl-2,2,5,5-tetramethyl-3-pyrroline-3-methyl methanethiosulfonate (MTSL, Adipogen). Excess spin label was removed by three rounds of centrifugation with ultrafiltration spin-columns (Amicon Ultracel 3K) at 3160g for 5-TR long peptides, and by three rounds of extraction with EtOAc for 3-TR long peptides. Peptides were lyophilized.

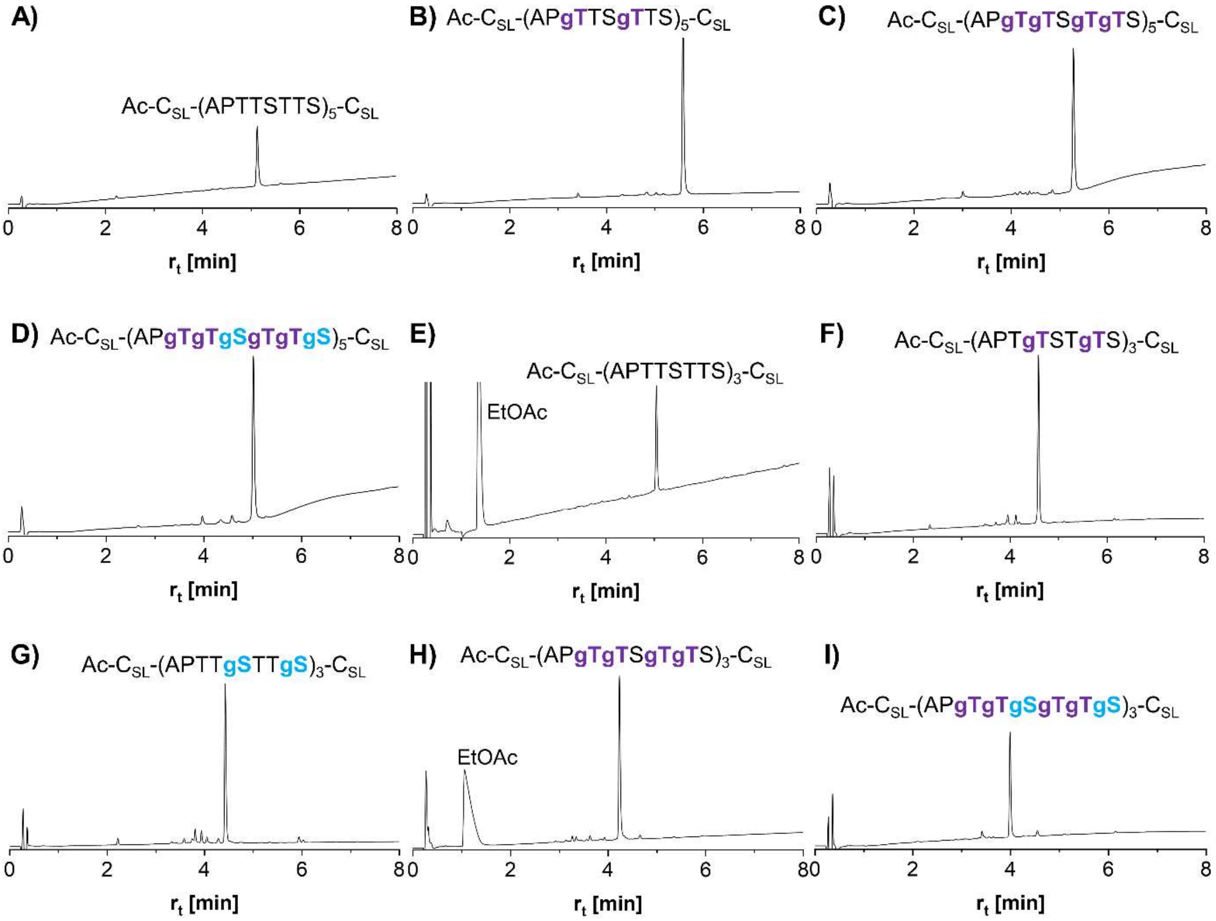

Figure S13. HPLC analysis of spin-labelled peptides **1' (A), 2' (B), 3' (C), 12' (D), 6' (E), 7' (F), 8' (G), 9' (H), 13' (I)**. Solvent A: 98.9% H_2O + 0.1% ACN + 0.1% TFA, solvent B: 98.9% ACN + 0.1% H_2O + 0.1% TFA. Gradient (A): 10-40% B in A, Gradient (B), (C), (D): 0-40% B in A, Gradient (E), (F), (H), (I): 05-50% B in A, Gradient (G): 07-50% B in A. λ = 210 nm.

8.2 Primary 4-pulse PELDOR data for peptides **1’**, **2’**, **3’**.

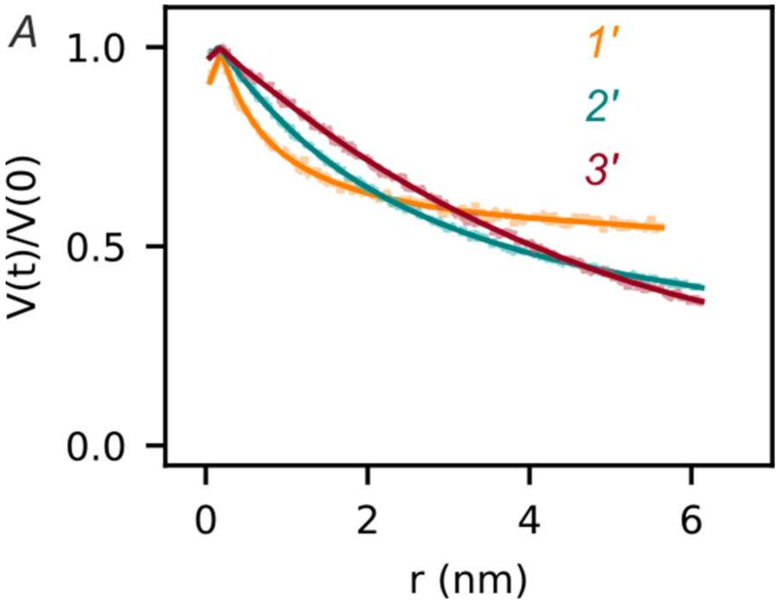

Figure S14. Primary 4-pulse PELDOR data for the 5-TR peptides are overlaid. For 1' and 2', the corresponding distance distributions are given in Fig 6. For 3', time-domain data reveal significantly longer distances, however, they cannot be calculated due to the difficulty in sufficiently prolonging the observed dipolar evolution time.

8.3 Continuous wave EPR

Continuous wave (CW) EPR spectroscopy measurements were performed in a Bruker ER422 SHQ 8304 (room temperature measurements) and TE102 (liquid nitrogen temperature measurements) resonators at X-Band (9.862 GHz) frequency, with a Bruker BER420 spectrometer upgraded with a Bruker ECS 041XG microwave bridge and a lock-in amplifier (Bruker ER023M); a modulation amplitude of 0.8 G and a modulation frequency of 100 kHz were used. The spectra were recorded with a receiver gain of 1e5 with a time constant of 20.48 ms and a conversion time of 80 ms. The magnetic field was swept from 3430 G to 3560 G in room temperature measurements and from 3250 G to 3490 G in low temperature measurements (114 K), the microwave attenuation was 20 dB. For all the measurements the samples with 50 uM concentration were prepared.

The CW-EPR of peptides (see Figure S15), with and without glycosylation, shows no indication of dipolar coupling, meaning the labels are more than 1.5 nm apart from each other.

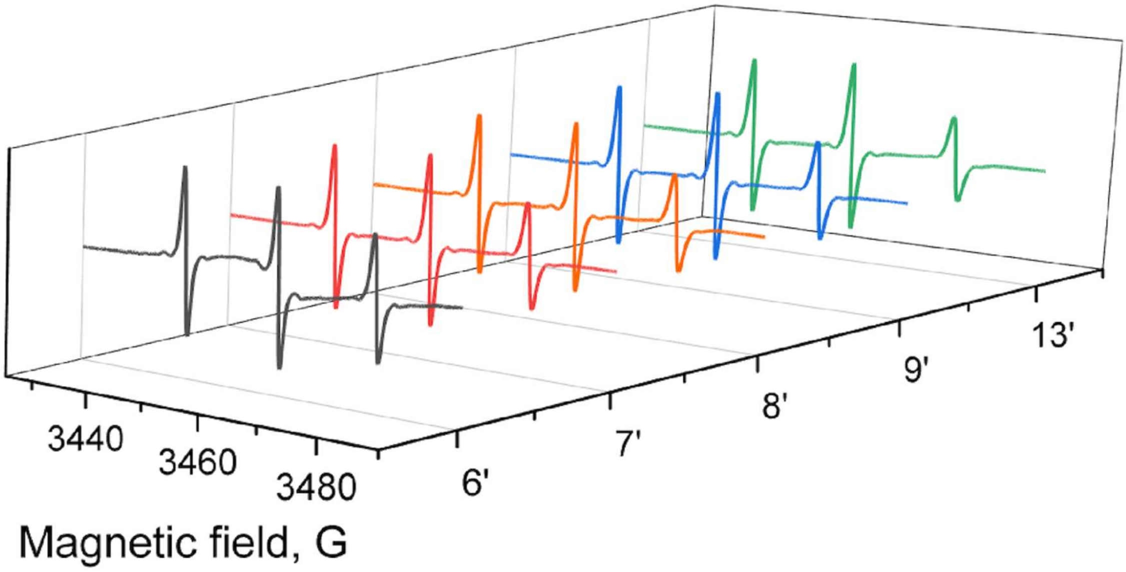

Figure S15. CW-EPR X-band spectra for short peptides with different quantity of glycosylation per repeating unit. Peptides were spin-labeled at cysteine residues engineered at the ends of the peptide.

The rotational correlation time of the paramagnetic reporter attached to the ends of the peptides increases (see Figure S16) in agreement with an increase of the molecular weight of the peptide upon glycosylation and subsequent slowing down of the global rotational dynamics of the peptide.

In general, the spectrum is the result of two competing effects on the correlation time. Firstly, an increase in molecular weight slows the global diffusional rotation of the peptide in solution, which in turn slows the motion of the labels attached to the peptide. This effect leads to an increase in the correlation time. Secondly, stiffening of the peptide backbone by glycosylation should enhance the local mobility of the terminally appended labels, because the contact with peptide residues would be hampered when labels can no longer bend back. This should result in an increase in mobility and a decrease in correlation time. It is noteworthy that both glycopeptides **7'** and **8'** possess identical molecular weights and identical degrees of glycosylation. However, glycopeptide **8'** exhibits a substantial increase in the rotational correlation time. This observation suggests that the peptide backbone of glycopeptide **8'** is more flexible than that of glycopeptide **7'**. This finding indicates that Ser(GalNAc) is less efficient in stiffening the backbone than Thr(GalNAc).

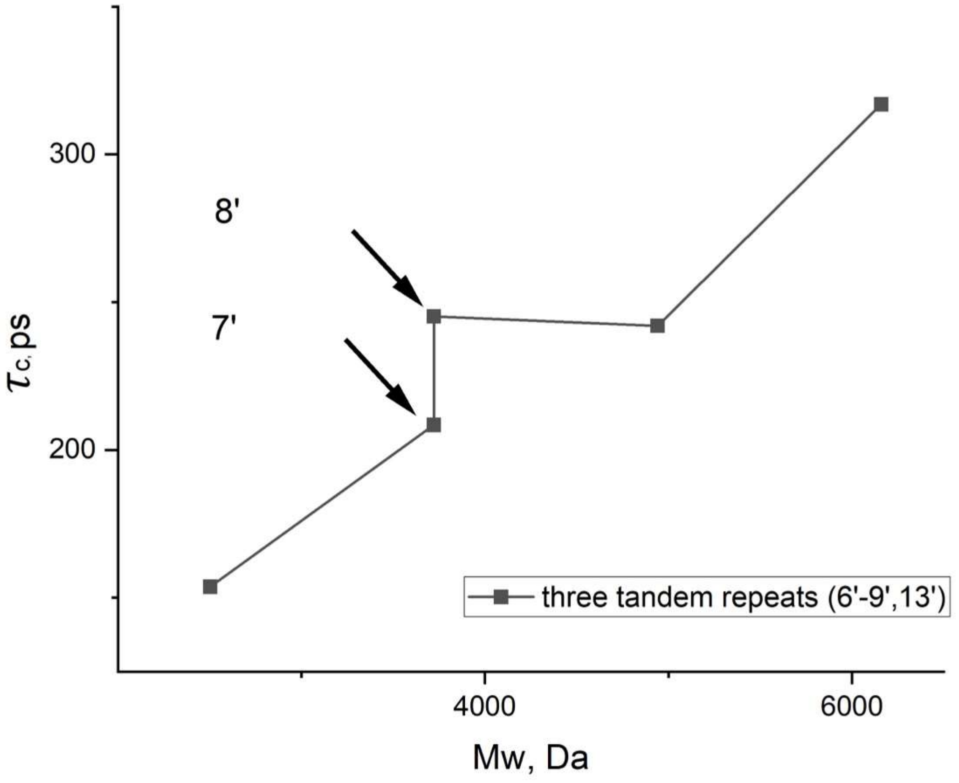

Figure S16. CW-EPR X-band spectroscopy data. Correlation time dependence on molecular weight and number of glycosylation sites for 26 amino acid long peptides **6’**, **7’**, **8’**, **9’**, **13’**.

9. Mass spectrometry data

9.1 ESI-MS spectra of peptides **4, 5, 11**

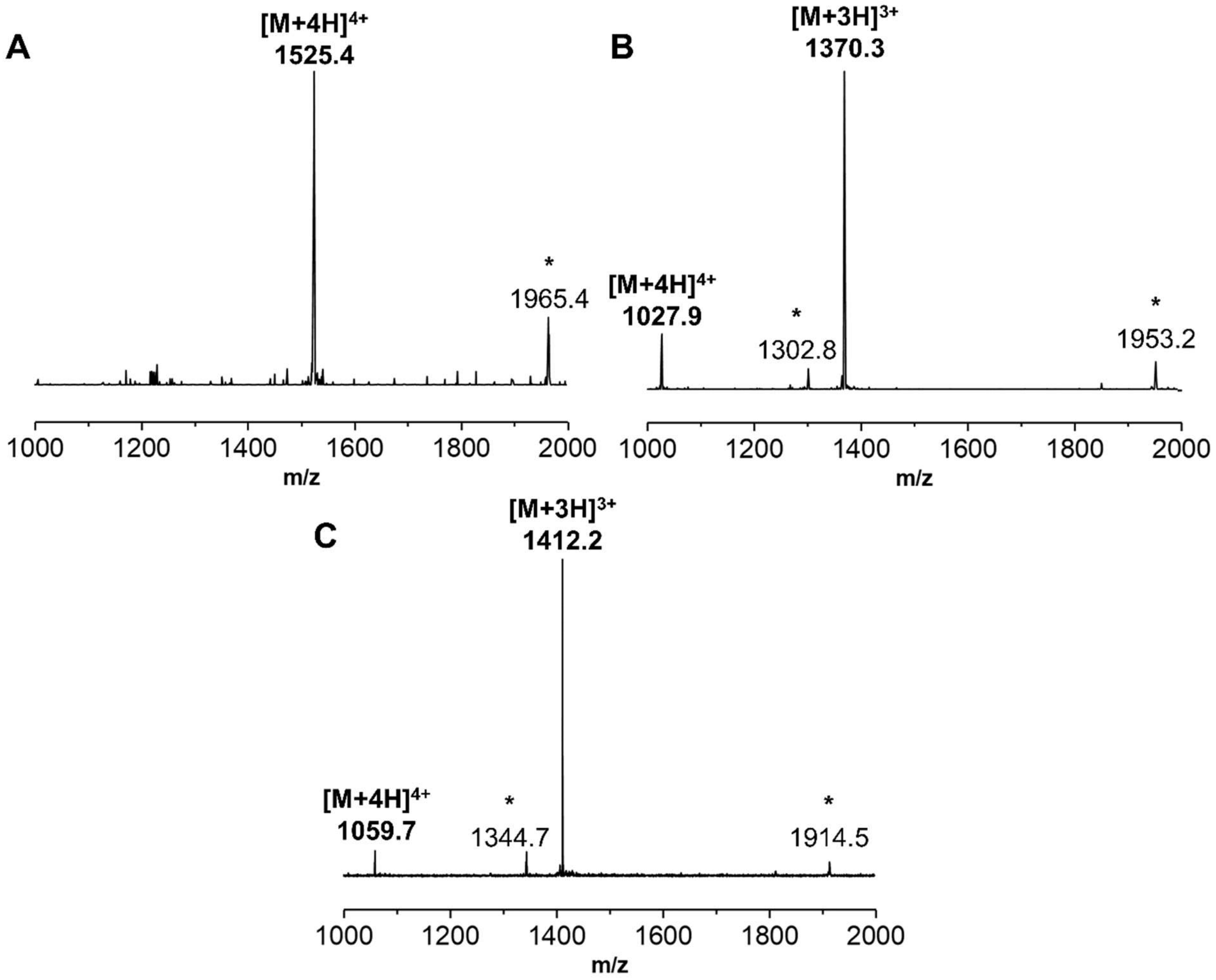

Figure S17. ESI-MS spectra of peptides **4** (**A**), **5** (**B**), **11** (**C**). Asterisks indicate mass peaks due to fragmentation during ESI-MS measurement.[1]

9.2 ESI-HRMS spectra of peptides **1-3, 12**

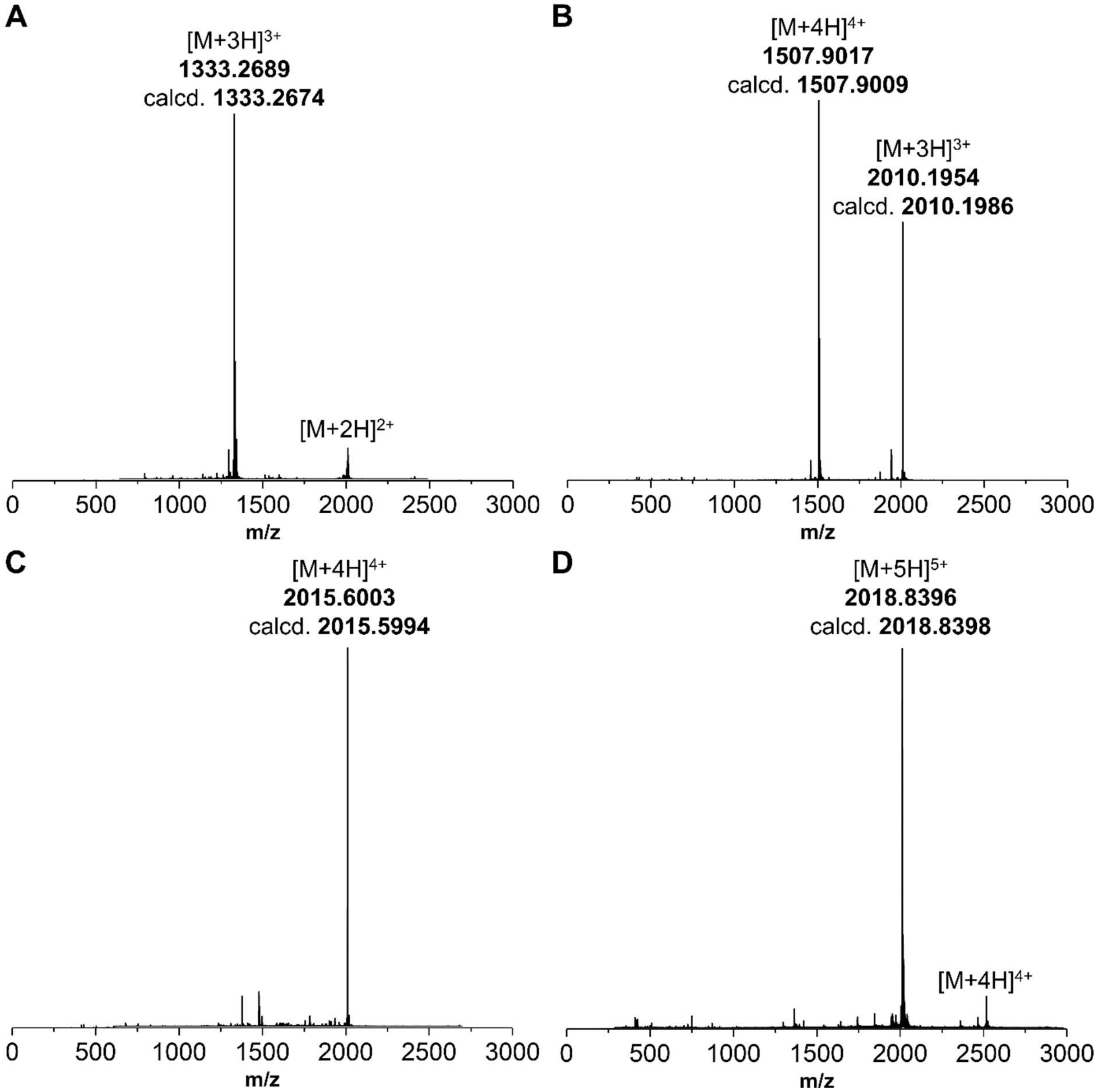

Figure S18. High Resolution ESI-MS spectra of peptides **1** (**A**), **2** (**B**), **3** (**C**), **12** (**D**).

9.3 ESI-HRMS spectra of peptides **6-9, 13**

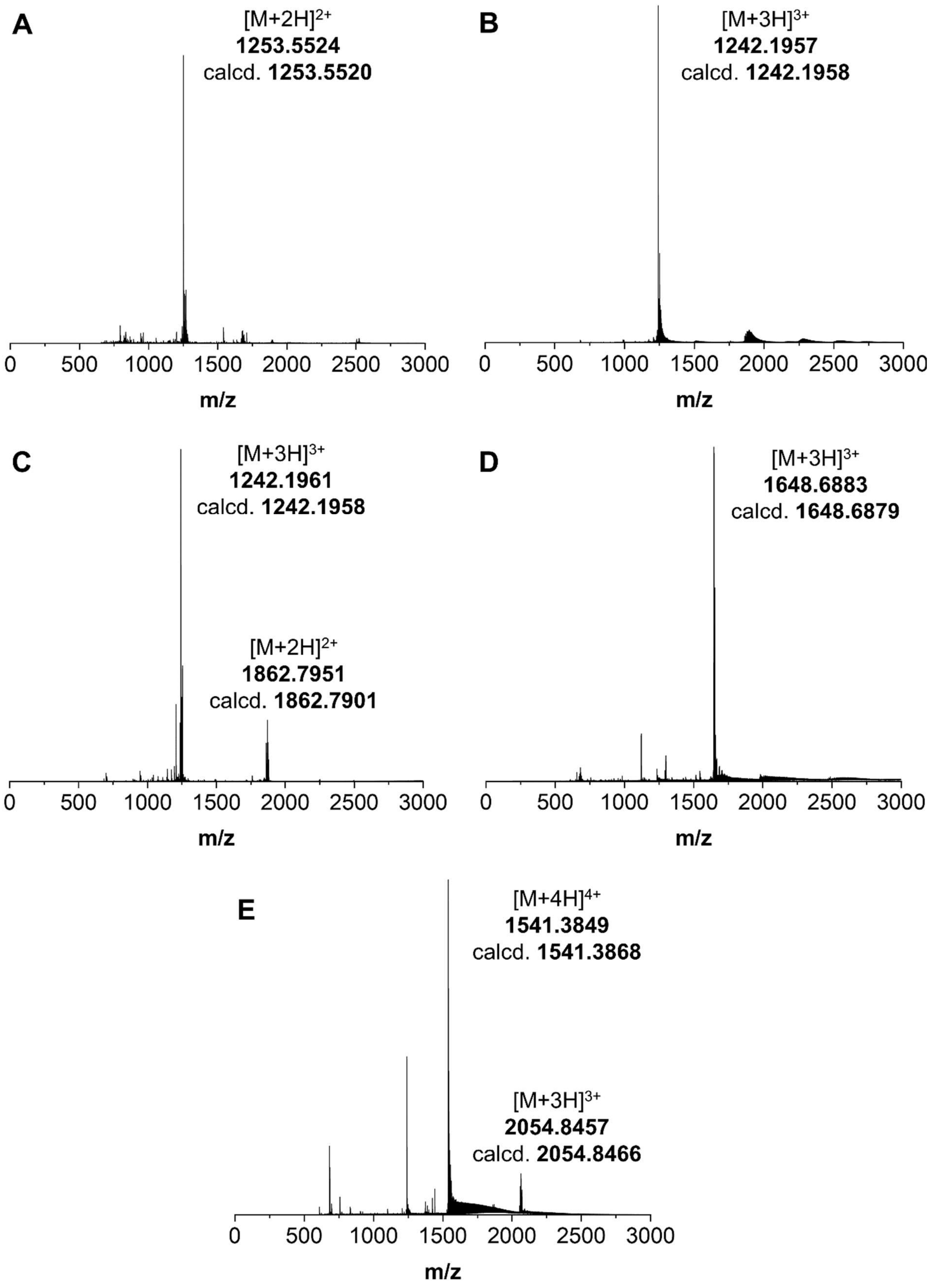

Figure S19. High Resolution ESI-MS spectra of peptides **6** (**A**), **7** (**B**), **8** (**C**), **9** (**D**), **13** (**E**).

10. Chemical structure of detergents Tween-20 and Triton X-100

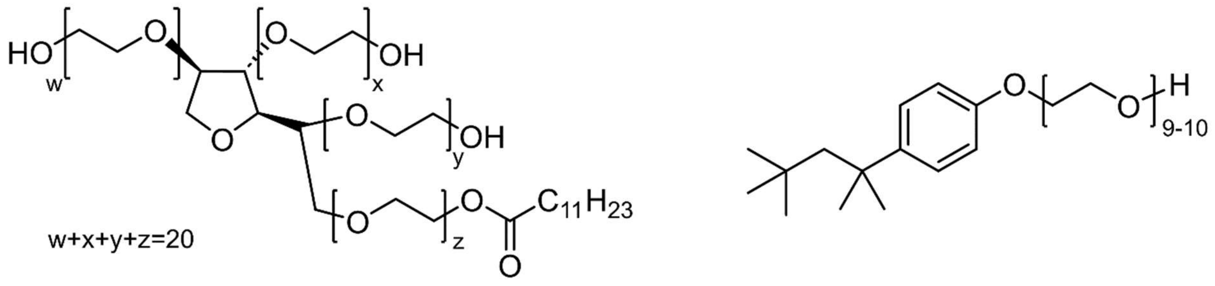

Tween-20 **Triton X-100**

Figure S20. Chemical structure of detergents Tween-20 and Triton X-100.

11. References

[1] A. Galashov, E. Kazakova, C. E. Stieger, C. P. R. Hackenberger, O. Seitz, *Chem. Sci.* **2024**, *15*, 1297-1305.
[2] M. Götze, L. Polewski, L. Bechtella, K. Pagel, *J. Am. Soc. Mass Spectrom.* **2023**, *34*, 2403-2406.
[3] L. Fabregas Ibanez, G. Jeschke, S. Stoll, *Magn Reson (Gott)* **2020**, *1*, 209-224.
